# Bicontinuous Phase Network Formed by Anti‐Plasticization Enhances Energy Storage Performance in Polyetherimide Dielectric Film

**DOI:** 10.1002/advs.202512343

**Published:** 2025-09-15

**Authors:** Xin Li, Le Zhou, Yao Xiao, Erxiang Xu, Taoyuan Yu, Mufeng Zhang, Minzheng Yang, Weibin Ren, Penghao Hu, Yang Shen

**Affiliations:** ^1^ School of Materials Science and Engineering State Key Lab of New Ceramics and Fine Processing Tsinghua University Beijing 100084 China

**Keywords:** anti‐plasticization, bicontinuous phase network, dielectric properties, high temperature energy storage, polymer composites

## Abstract

Polymer dielectrics display high breakdown strength (*E*_b_) and larger power density, rendering them an indispensable component in electronic energy storage applications. Nevertheless, the discharged energy density (*U*_d_) of polymer dielectrics is limited by relatively low dielectric constant (ε_*r*_) and sharply decreases in *E*_b_ at elevated temperatures. The simultaneous improvement in both *E*_b_ and ε_*r*_ is highly desired, but the inverted relationship requires urgent resolution. Herein, the study introduces several plasticizers with low content into polyetherimide (PEI) matrix to fabricate composites. The formation of a bicontinuous phase network in polymer matrix is achieved through anti‐plasticization. Owing to the discrepancy in polarizability, the network can achieve an electric field redistribution and interface polarization. It is composed of a dielectric phase (bear a lower electric field) and an insulation phase (bear a higher electric field), resulting in a concomitant enhancement on both ε_*r*_ and *E*_b_. A relatively high *U*_d_ of 4.88 J cm^−3^ accompanied by *η* = 90% and charge‐discharge cycle stability up to 10^5^ cycles at 150 °C are achieved in the composite content with 3 wt.% of butylsuccinic anhydride. This work presents a promising strategy for decoupling the inverse relationship and fabricating applicable high‐temperature polymer dielectrics through phase structure modulation.

## Graphical abstract

A bicontinuous phase network in PEI matrix is achieved through anti‐plasticization. The network is composed of a dielectric phase and an insulation phase, respectively, and realized enhancement on both breakdown strenght and dielectric permittivity, resulting in a high discharged energy density with 10^5^ charge–discharge cycle stability. The thermal and mechanical properties of the composites are also preserved.

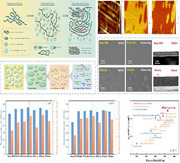

## 1 Introduction

Polymer dielectrics are one of the excellent storage media of electrostatic film capacitors due to their intrinsic high breakdown strength (*E*_b_) and charge‐discharge efficiency (*η*), great flexibility, low cost, excellent processability, and so on. In contrast to other capacitor counterparts such as ceramics and electrolytic capacitors, electrostatic film capacitors exhibit ultrafast charging‐discharging speed, ultrahigh power density, and longer cycle lifetime. Consequently, they are facilitated to be applied in advanced electrical and electronic systems (such as wind turbine generators, heat pump system, gas and oil exploration, and so on).^[^ ^1^, ^2^, ^3^ ^]^ However, most of these applications necessitate capacitors to endure extreme temperatures (exceeding 150 °C). This requirement poses a significant challenge for the use of polymer dielectrics because of their decreased discharged energy density (*U*_d_) caused by the deteriorated *E*_b_ and *η* at elevated temperatures. In principle, *U*_d_ can be mainly determined by both the dielectric constant (ε_*r*_) and *E*_b_, but there is a paradoxical relationship existing between these two factors, which limits the energy storage capability of monothetic materials.^[^ ^4^, ^5^ ^]^

A number of strategies have been proposed to decouple the inverse relationship, particularly at elevated temperatures, according to the emerging demand for high‐temperature capability in recent years. For instance, the incorporation of suitable content of inorganic fillers with surface modification can augment the *E*_b_ due to the inducement of deep traps, as well as enhance ε_*r*_ by the generation of interfacial polarization and the modulation of the configuration and mobility of polymer chains.^[^ ^6^, ^7^ ^]^ Furthermore, 1D and 2D fillers with specific orientations are employed to enhance both *E*_b_ and ε_*r*_ concurrently, through the inhabitation of electric tree growth and the introduction of interfacial polarization.^[^ ^8^ ^]^ Nevertheless, the introduction of inorganic fillers is very prone to aggregation and inhomogeneous composition, which leads to defects in material, thereby impairing the apparent performance of polymer composites and increasing difficulties of large‐scale production.^[^ ^9^ ^]^ The design of all‐organic dielectric polymers, including the rational design of molecular structures and the introduction of additional organic components, is also proposed.^[^ ^10^ ^]^ Firstly, the molecular approaches can achieve higher glass transition temperature (*T*_g_) and energy bandgap (*E*_g_) by introducing rigid noncoplanar segments into aromatic polymers or designing alicyclic polymers. But the relatively low ε_*r*_ greatly limits the enhancement on energy storage performance.^[^ ^11^ ^]^ Dipolar glass polymers prepared via the introduction of polar groups into the polymer chain have also been proposed to enhance dipole polarization. However, monitoring the quantity and size of the introduced dipoles is crucial to prevent an increase in dielectric loss and a sharp decline in *η* at elevated temperatures.^[^ ^12^ ^]^ The cost implications of molecular structure design should also be taken into account. Secondly, the doping approaches represent a direct and potent way to enhance high‐temperature performance by leveraging the respective advantageous properties of the added component in conjunction with the matrix. For instance, the introduction of molecular semiconductors can form deep carrier traps to generate strong binding forces with free electrons, leading to the suppression of leakage current and an increase in *E*_b_.^[^ ^13^, ^14^ ^]^ Organic molecules, such as rigid aromatic molecules, can form a dense stacking structure by electrostatic interaction forces to affect energy storage capability.^[^ ^15^ ^]^ But the paradox of mutually exclusive constraints on *E*_b_ and ε_*r*_ continue to affect the overall performance of these composites. Therefore, improving both *E*_b_ and ε_*r*_ simultaneously in all‐organic composites with straightforward methods is a highly promising avenue for scalable, continuous, and large‐scale production.

**Figure 1:**
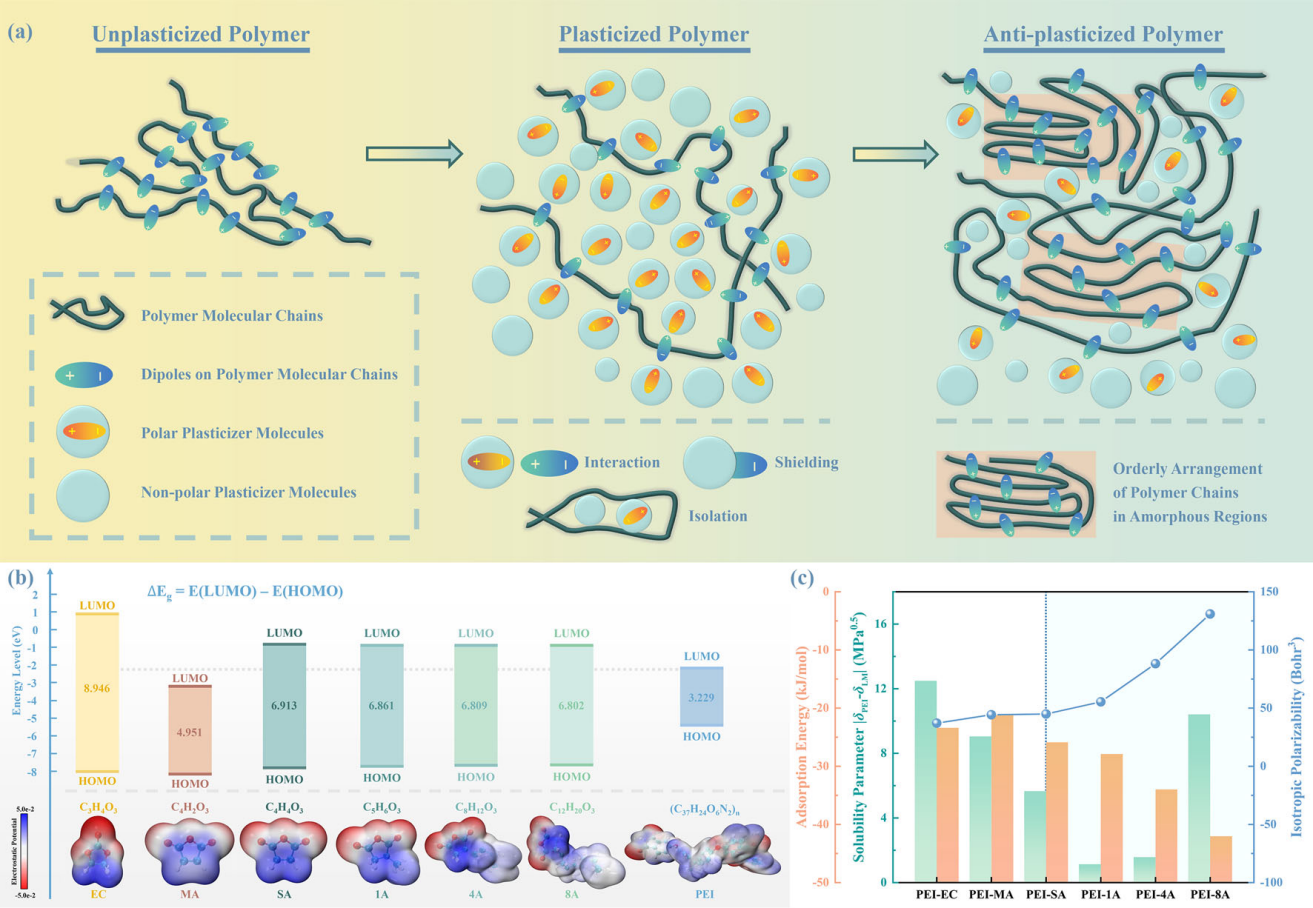
a) Schematic illustration of the interaction between polymer and plasticizer molecules. b) Schematic chemical structures, electrostatic potential distribution, and bandgaps of plasticizer molecules and polyetherimide. c) Adsorption energy, solubility parameter between components, and isotropic polarizability of plasticizer molecules.

Generally, plasticizers with a low weight (LMs for short) and a limited number of polar groups are employed as an additive within the polymer matrix to make composites softer and more pliable. As shown in **Figure** **1**a, a plasticizer typically comprises two structural components, polar and non‐polar. The polar component contains polar functional groups, while the non‐polar component is constituted by alkyl chains. Each of the two molecular segments can play a role in isolation, increasing the distance between adjacent chains and hindering their interaction.^[^ ^16^ ^]^ Moreover, the polar segment is also capable of reversibly binding with the polymer, because of the interaction between the dipoles present on both the polymer chain and the molecule. The non‐polar segment can contribute to the shielding of other polar sites on the polymer chain. Consequently, some specific types of LMs can significantly influence the mobility of chains by modulating the interaction modes without the creation of new chemical bonds, thus offering a promising avenue for the improvement of ε_*r*_.^[^ ^17^ ^]^ Nevertheless, only a moderate degree of plasticization will prove beneficial for the final performance. There is poor mechanical strength and lower *T*_g_ at a higher plasticization degree, which suppresses the improvement of *E*_b_.^[^ ^18^ ^]^ It has been found that at low concentrations of LMs, the molecules can be envisaged as being “bound” to the polymer chains. This binding can restrict the secondary relaxation (*β* relaxation) and augment Young's modulus by suppressing the local dynamics and reducing the local free volume, thereby giving rise to an anti‐plasticization behavior.^[^ ^19^ ^]^ Normally, the amorphous areas in polymer have more free volume, and LMs preferentially situate in these regions. For anti‐plasticization, a novel redistribution of the configurations of polymer chains is permitted due to the attachment of a small quantity of LMs to the polymer, resulting in an observed increase in order within the amorphous areas of non‐crystalline polymers. The aforementioned compactly lined‐up areas are capable of imparting additional rigidity to the polymer, offering potential to resolve the paradox of ε_*r*_ and *E*_b_ in all‐organic composites.^[^ ^20^ ^]^

Herein, six LMs, i.e., succinic anhydride (SA with 1 wt.%), maleic anhydride (MA with 5 wt.%), ethylene carbonate (EC with 3 wt.%), methylsuccinic anhydride (1A with 3 wt.%), butylsuccinic anhydride (4A with 3 wt.%), and n‐octylsuccinic anhydride (8A with 3 wt.%), are selected as fillers to verify the effect of different types of plasticizers on the dielectric properties via anti‐plasticization in PEI‐based composites. The chosen optimal contents are related to the properties of composites. The synthesized PEI‐LMs composites exhibit a bicontinuous phase network, which is induced by anti‐plasticization as well as the liquid‐liquid phase separation that occurs due to the difference in melting points between the two components. The rich‐phases of polyetherimide and LMs separately act as insulation phase and dielectric phase, respectively, which results in a collaborative improvement of *E*_b_ and ε_*r*_ at 150 °C in all these all‐organic composites due to an electric field redistribution and interface polarization. The introduction of butylsuccinic anhydride, designated PEI‐4A, exhibits a superior *U*_d_ of 4.88 J cm^−3^ accompanied by *η* = 90% with a high ε_*r*_ of 3.75 (1 kHz) and *E*_b_ of 617 MV m^−1^ @ 150 °C. Moreover, the thermal and mechanical properties of the composites have been investigated to ascertain the benefits of anti‐plasticization. Additionally, all the PEI‐LMs demonstrate stable performance during long‐term charging–discharging cycling, indicating a potential application for electrostatic capacitors at elevated temperatures.

## 2 Results and Discussion

### 2.1 Preparation and the Establishment of Bicontinuous Phase Network

As illustrated in Figure 1b, all of the introduced LMs exhibit suitable energy levels with benefits for *E*_b_, as well as possessing polar groups to improve ε_*r*_. In addition to MA, the LUMO levels of the fillers are higher than that of the matrix, while the HOMO levels are lower, which can act as the anti‐traps to scatter the charge carries and impede their transportation. MA, on the other hand, exhibits lower LUMO and HOMO levels, signifying an enhanced capacity to trap electrons.^[^ ^21^ ^]^ Moreover, the distribution of electrostatic potential indicates that the polar groups of LMs carry negative charge (highlighted in red), while the phenyl groups bonded to imide groups are positively charged (highlighted in blue). The electrostatic potential range of PEI and six kinds of LMs is also shown in Figure S1 (Supporting Information). This indicates the potential interaction location is established due to the electrostatic force. Moreover, the positive electrostatic potential can attract electrons and function as electron trap sites, while the negative electrostatic potential can demonstrate attraction to holes, leading to the formation of hole traps. Thus, the incorporation of LMs will result in the presence of additional strong, localized deep charge traps, which will improve the electrical performance and affect the local electronic states of PEI chains due to the electrostatic interaction between PEI chains and LMs.^[^ ^22^ ^]^ In order to ascertain the miscibility of the polymer/plasticizer blend, Hildebrand solubility parameters and adsorption energy were calculated and shown in Figure 1c. The calculated Hildebrand solubility parameter for EC is 32.19 MPa^0.5^, which is in excellent agreement with the reported values in literatures, providing compelling evidence for the accuracy of the calculated results.^[^ ^23^ ^]^ The discrepancy between δ_PEI_ and δ_LM_ with the minimum value can be employed to determine the compatibility and the dispersity for LMs. As shown in Figure 1c, PEI‐SA possesses a smaller δ_PEI_‐δ_LM_ value, representing superior compatibility compared to MA and EC, while 1A and 4A display comparatively lower values than SA. Moreover, the adsorption energy is an index that can measure the adsorption intensity in the adsorption process. The greater the negative value of the energy is, the stronger the intensity of the interaction is.^[^ ^24^ ^]^ Thus, the lower adsorption energy typically suggests a stronger interaction between PEI and LMs, with SA and 8A exhibiting the better results. Usually, the interaction is inversely proportional to the difference in solubility parameters.^[^ ^25^ ^]^ However, PEI‐8A exhibits a reduced compatibility but a stronger force, which can be attributed to the enhanced shielding effects resulting from the too‐long alkyl chains.^[^ ^26^ ^]^ The improved compatibility and efficacious polymer‐LMs interaction are conducive to the anti‐plasticization. Additionally, the isotropic polarizability of LMs has also been calculated and is illustrated in Figure 1c. The higher value can lead to a stronger dipole‐dipole interaction, i.e., SA has a higher value of 44.93 Bohr^3^ than EC (37.11 Bohr^3^) and MA (44.29 Bohr^3^), which is consistent with the aforementioned outcomes.

**Figure 2:**
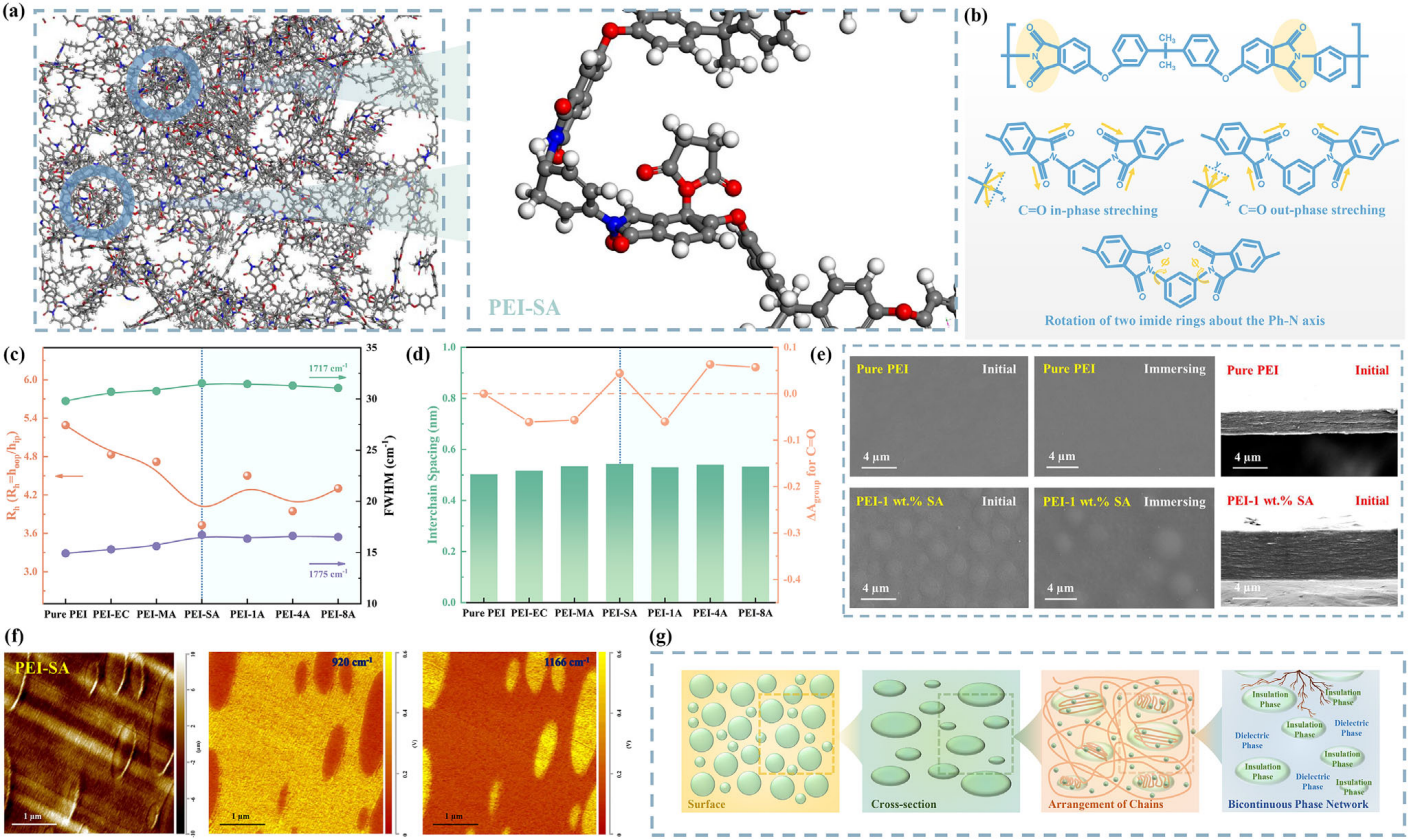
a) MD results of the interaction between PEI and SA. b) Vibration modes of C = O of PEI. c) The height ratio (R_h_) and FWHM of the two C = O bands of samples. d) Calculated interchain spacing determined by XRD data and ΔA_group_ for C = O determined by Raman spectra. e) Surface and cross‐section SEM images of pristine PEI and PEI‐SA. f) Simultaneously measured topography (left) and AFM‐IR chemical maps with irradiation by a laser at 1166 cm^−1^ (middle) and 920 cm^−1^ (right), respectively. g) Schematic illustration of the formation of multilayer structure network.

Molecular dynamics (MD) simulations between LMs and PEI are conducted, and the pertinent results are presented in **Figure** **2**a and Figure S2 (Supporting Information). The opposing charged regions between the polar groups in LMs and the phenyl groups in PEI, are preferentially bound to the LMs, which can affect the rotation about the Ph‐N axis.^[^ ^27^ ^]^ As shown in Figure 2b, there are two types of C = O stretching bands in cyclic imides for PEI, which are located at 1778 and 1725 cm^−1^, respectively.^[^ ^28^ ^]^ The former with lower intensity is attributed to the C = O in‐phase stretching, where the dipole moment is induced by the symmetric stretching and aligns along the x‐axis (defined by the Ph‐N bond). The latter with higher intensity is referred to as the C = O out‐of‐phase stretching where the dipole moment is caused by the asymmetric stretching and aligns along the y‐axis. The “net dipole moment” is determined by summing the C = O out‐of‐phase stretching in all the imide groups, and it is influenced by the absorption intensity. Moreover, the rotation angle (Φ) of the imide rings about the Ph‐N axis can also reflect the magnitude of the net dipole moment due to its impact degree on coplanarity. Normally, there is a beneficial intramolecular interaction between the oxygen long‐pair electrons and the π electrons of the phenylene group in pristine PEI (where Φ > 45°). However, the incorporation of LMs can replace it with an intermolecular interaction, leading to promoting rotation about the Ph‐N axis and an increase in coplanarity, to maximize the binding to polymer chains. The intensity ratio *R* of these two bands can be used to explain the change and the less coplanarity, i.e., a decreasing *R* indicates a stronger interaction. The FTIR spectra (Figure S3, Supporting Information) reveal that no additional bonds formed in the composites. According to Figure 2c, all PEI‐based composites exhibit reduced *R* values, with PEI‐SA and PEI‐4A displaying a comparatively lower ratio of 3.73 and 3.95, respectively. In addition, the broadening of the width at half‐height (FWHM) for the two bands is primarily attributed to the introduction of dipoles from LMs, which is beneficial for the improvement of dielectric constant. The interchain spacing *d* calculated from XRD data (Figure S4, Supporting Information) increases from 0.502 nm for PEI to 0.543 nm for PEI‐SA and 0.540 nm for PEI‐4A (Figure 2d). The higher polarizability may result in a stronger interaction, which is in line with the FTIR and XRD results. Whereas, the anomalous behavior observed in PEI‐1A and PEI‐8A may be associated with intensified shielding effects resulting from the too short or too long alkyl chains of LMs, which restrict the rotation of groups and the increase in chain spacing. Furthermore, Raman spectroscopy is utilized to monitor alterations in C = O groups, yet it remains the similar major bands across all samples (Figure S5, Supporting Information).^[^ ^29^ ^]^ The alterations in relative areas ΔA_group_ of C = O bands at 1780 cm^−1^ in comparison to the phenyl groups at 1620 cm^−1^ can be indicative of the variation in concentration, and the relative values are of particular significance, due to the influence of unavoidable interactions on the vibration of phenyl groups. As seen in Figure 2d, PEI‐SA, PEI‐4A, and PEI‐8A exhibit positive ΔA_group_, which are associated with the larger interchain spacings and more dipoles from the LMs. The lower ΔA_group_ for PEI‐1A, PEI‐MA, and PEI‐EC may be caused by the suppression of C = O vibration due to the limited free volume and the shielding of dipoles. The larger interchain spacing and stronger interaction are beneficial for the improvement of polarization and anti‐plasticization.

The surface and cross‐sectional morphologies of the PEI and PEI‐LMs composites are shown in Figure 2e and Figure S6 (Supporting Information). Compared with pristine PEI, all composites exhibit pronounced phase separations, but the sizes of which are discernibly different. However, there are no notable differences observed in the cross‐sectional images, indicating that the formed rich phases have a relatively thin profile. To further identify the composition, the chemical mapping was conducted using an infrared laser at wavelengths of 920 and 1166 cm^−1^. These wavelengths relate to the characteristic bands of the vibration of the cyclic anhydride ring in LMs and the C‐N stretching in PEI, respectively (Figure S7, Supporting Information).^[^ ^30^, ^31^ ^]^ As illustrated in Figure 2f, the PEI‐rich phase is dispersed as droplets within the SA‐rich matrix in PEI‐SA composite. Following a 20‐day immersion in n‐hexane to remove LMs from the surface, the phase separation becomes more pronounced and the droplets display a reduced erosion rate (Figure 2e), further substantiating the coexistence of two rich phases. The observed morphology is in accordance with the increased orderliness of the amorphous regions caused by anti‐plasticization, where LMs with low doping content tend to locate at the disordered region and increase the interchain spacing (Table S1, Supporting Information). Concurrently, the disparity in melting points between LMs and PEI (Table S2, Supporting Information) can also cause a liquid‐liquid phase separation where PEI initially solidifies during the cooling process to form the droplets.^[^ ^32^ ^]^ Moreover, the distribution of droplets in the topography is observed to be perpendicular to the thickness direction of the samples, exhibiting a disk‐like morphology. According to the equation ε_1_ *E*_1_ =ε_2_ *E*_2_, it can be seen that the phase with higher dielectric constant, like LMs‐rich phase, can bear a lower electric field, while another phase with lower dielectric constant, such as PEI‐rich phase bears a higher electric field.^[^ ^33^ ^]^ Thus, owing to the discrepancy in polarizability between LMs and PEI, the PEI droplets can act as an insulation phase to absorb most of the applied electric field and prevent the extension of electric trees. The establishment of a bicontinuous phase network has resulted in an electric field redistribution. Meanwhile, the LMs‐rich phase can serve as dielectric phase to significantly enhance the dielectric constant.^[^ ^34^ ^]^ These designations are considered relative definitions that do not alter the inherent nature of the materials as insulators. The ordered arrangement of chains in PEI‐rich phase can also contribute to maintaining stiffness of the composite. Moreover, the additional interfacial polarization and the blocking effect at interfaces resulting from the droplet conformation may also contribute to the improvement of ε_*r*_ and *E*_b_. The schematic illustration of bicontinuous phase network formation is shown in Figure 2g. Whereas, the presence of a greater number of smaller droplets within PEI‐1A, PEI‐8A, and PEI‐MA resulted in a reduction in impedance along the electrical breakdown path. The lower difference between δ_PEI_ and δ_1A_ leads to an almost homogeneous morphology, and the strengthened shielding effects in PEI‐8A can restrict the dipole‐dipole interaction in the orderly arranged regions. Furthermore, the optimal content of MA up to 5 wt.% can also reduce the area of these ordered regions by distributing in more amorphous region and weaken the anti‐plasticization with more doping content compared to other composites.

### 2.2 Thermal and Mechanical Properties

**Figure 3:**
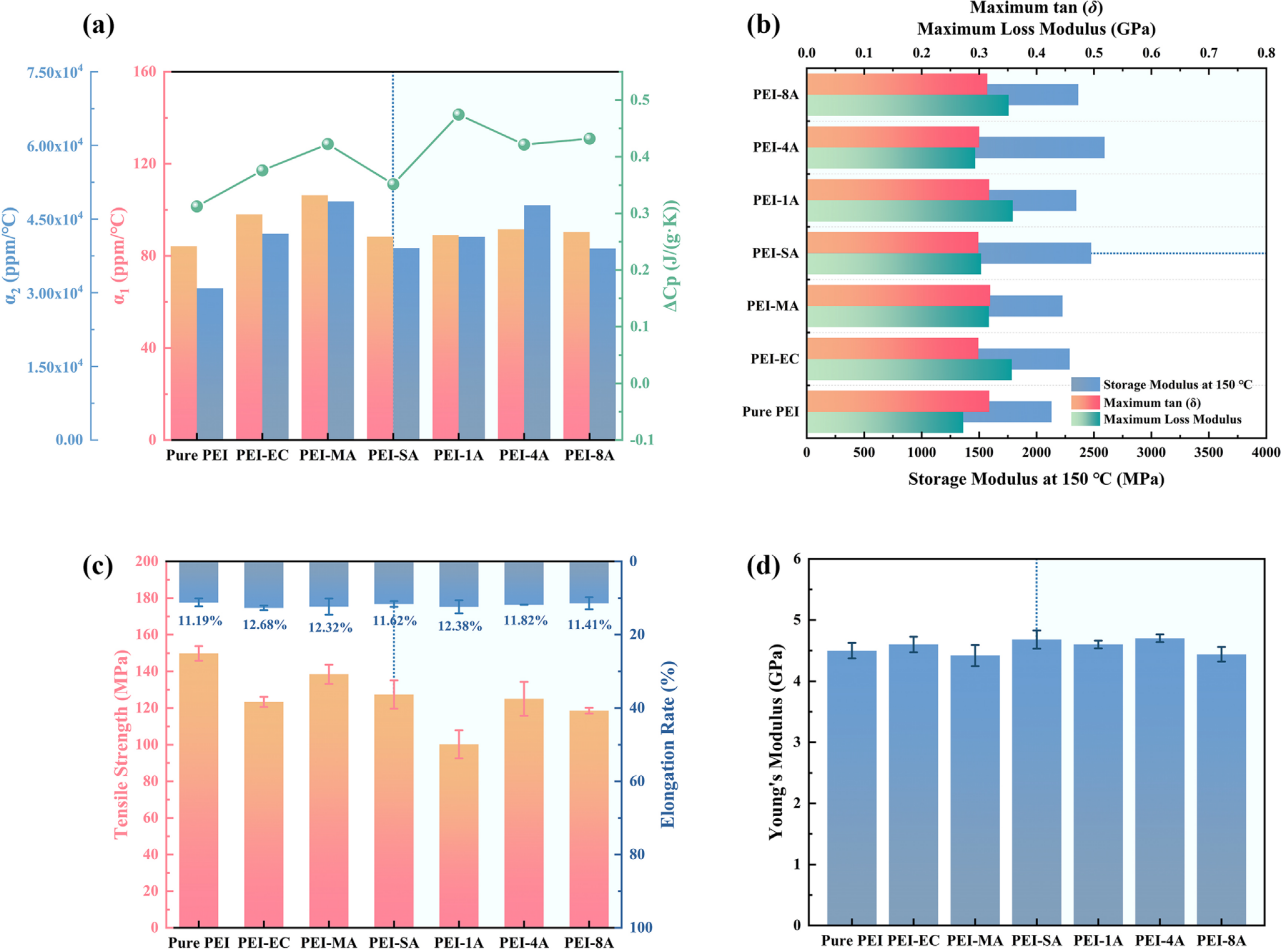
a) Thermal diffusion coefficient before and after glass transition α_1_ and α_2_ determined by TMA curves and *ΔC*_p_ determined by DSC curves. b) Dynamic mechanical analysis (DMA) results of samples. c) Tensile strength and elongation rate values of samples from tensile test. d) Young's modulus values of samples from nano‐indentation test.

Owing to the unusual shifts in *T*_g_ and stiffness resulting from anti‐plasticization, an examination of the thermal and mechanical properties is essential to further clarify the mechanisms governing the dielectric properties.^[^ ^35^ ^]^ It is evident that the weight loss (2.5‐5%) of the synthesized composites is primarily confined to the temperature range of 300–500 °C, as observed in the TGA and DTG curves (Figure S8, Supporting Information). And there is no other apparent weight loss stage, indicating an excellent compatibility between LMs and PEI matrix. As illustrated in **Table** **1**, the temperature at which 5% weight loss in PEI, PEI‐SA, PEI‐4A and PEI‐EC are all within the range of 472–484 °C, demonstrating an excellent thermal stability due to their sparse phase separation configuration. Moreover, the temperature of maximum rate of mass loss can be identified by the peak location in the DTG curve. It is noteworthy that PEI‐SA, PEI‐4A, and PEI‐EC all exhibit higher values. The larger droplets of PEI can lead to a more compact segment packing, resulting in the enhanced thermal stability.^[^ ^36^ ^]^ The *T*_g_ of the composites has also been investigated by DSC, TMA, and DMA, which presents a similar trend, and a lower decrease is observed for the addition of SA, 4A and EC. Furthermore, the glass transition of all samples takes place at a single temperature, indicating a homogeneous dispersion. The DSC curves and the corresponding *T*_g_ for composites with different content of LMs (Figure S9 and Table S3, Supporting Information) demonstrate that an excess of LMs will segregate into small clusters of molecules owing to the strengthened plasticizer‐plasticizer attractions at elevated concentration, thereby converting the mechanism toward plasticization.^[^ ^37^ ^]^ At the same time, some LMs fail to fulfil the role of isolation, leading to a reduction in chain spacing but a lower *T*_g_ due to the lack of anti‐plasticization. More importantly, the Δ*C*_p_ values of composites as summarized in **Figure** **3**a, illustrates a change in the degrees of freedom that occurs when the polymer transitions from a highly self‐entangled state to an extended state.^[^ ^38^ ^]^ All the samples exhibit a higher value than that of pristine PEI, which is caused by the weakened polymer‐polymer interaction resulting from the addition of LMs. Moreover, the ordered arrangement of chains in droplets exhibits a heightened interaction between chains, and the diverse morphologies lead to the observed discrepancy in Δ*C*_p_ between different types of composites. The introduction of SA and 4A results in lower values of 0.35 and 0.42 J g^−1^K^−1^, respectively, indicating a strong interaction that is beneficial for the improvement of *E*_b_. The relatively higher Δ*C*_p_ values for PEI‐1A, PEI‐8A, and PEI‐MA are related to their morphology, which comprises a greater proportion of amorphous regions. The calculated thermal expansion coefficients (CTE) before and after glass transition α_1_ and α_2_ are illustrated in Figure 3a (see TMA curves in Figure S10, Supporting Information). The thermal expansion of polymers is predominantly governed by intermolecular forces, and electronic devices demand polymer dielectric with a lower CTE.^[^ ^39^ ^]^ Furthermore, the CTE is inversely proportional to the Young's modulus.^[^ ^40^ ^]^ Owing to the substitution of polymer‐LMs interactions in composites, there is a slight improvement in CTE. The α_1_ of PEI‐SA and PEI‐4A is 88.43 and 91.58 ppm/°C, respectively, in comparison to the value of 84.22 ppm/°C for pristine PEI. The minor variation suggests that there is minimal deterioration in dielectric performances.

**Table 1:** Thermal data obtained from TGA, DSC, TMA and DMA analysis of samples.

	T_5%_	T_max_	T_g_ (DSC)	T_g_ (TMA)	T_g_ (DMA)		
					T_g_ E’	T_g_ E’’	T_g_ (tan δ)
Pristine PEI	477.95	547.79	216.92	211.87	202.06	205.37	209.18
PEI‐EC	478.47	543.60	206.94	210.38	202.95	204.85	206.05
PEI‐MA	436.98	531.42	194.79	209.35	190.62	191.52	192.81
PEI‐SA	484.88	548.24	211.06	210.49	205.48	206.48	208.78
PEI‐1A	418.66	541.06	204.82	204.79	196.78	197.48	198.08
PEI‐4A	472.70	543.06	205.64	205.02	202.45	203.45	207.05
PEI‐8A	395.97	538.90	190.38	199.55	186.42	187.82	188.72

DMA tests have been conducted to assess the enhancement in mechanical strength caused by anti‐plasticization (see curves in Figure S11, Supporting Information). Wherein, the storage modulus (*E′*) is frequently associated with stiffness and represents a measure of the sample's elastic behavior. In contrast, the loss modulus (*E″*) is defined as the capacity to dissipate energy that is related to the internal molecular mobility. The damping factor, tan (δ), is the ratio of *E″* and *E′*, which can be used to determine the occurrence of molecular mobility transitions and indicate how the materials lose energy due to the internal friction and molecular arrangements.^[^ ^41^ ^]^ As shown in Figure 3b, the *E′* at 150 °C exhibits a slight increase from 2130.6 MPa for PEI to 2475.7 MPa for PEI‐SA and 2591. 4 MPa for PEI‐4A, respectively, indicating an improvement in stiffness and an enhanced interface bonding due to the anti‐plasticization. The increase in *E″* signifies the limitation of chain mobility and the restricted free volume in ordered arrangement regions. And the enlargement of these areas is beneficial for the lower *E″*, as evidenced in PEI‐SA and PEI‐4A.^[^ ^42^ ^]^ Moreover, the lower tan (δ) for composites can be attributed to the constraint of secondary relaxation and an increase in energy dissipation.^[^ ^43^ ^]^ The relative tensile strength and elongation of composites are displayed in Figure 3c (see the tensile strain curves in Figure S12, Supporting Information). The decrease in strength and increase in elongation are in line with the macroscopic plasticization due to the changes in intermolecular forces. However, the microscopic phase separation caused by anti‐plasticization can counteract further degradation in performance. Nanoindentation tests have also been conducted, and shown in Figure 3d, reveals that the Young's modulus is slightly improved, e.g. 4.68 GPa for PEI‐SA and 4.70 GPa for PEI‐4A compared to PEI (4.50 GPa). In conclusion, the configuration and distribution of the formed disk‐like shape phase are beneficial in maintaining stiffness and preventing a significant decline in mechanical properties, thereby enhancing the electrostatic energy storage performance of the composites.

### 2.3 Dielectric Properties

**Figure 4:**
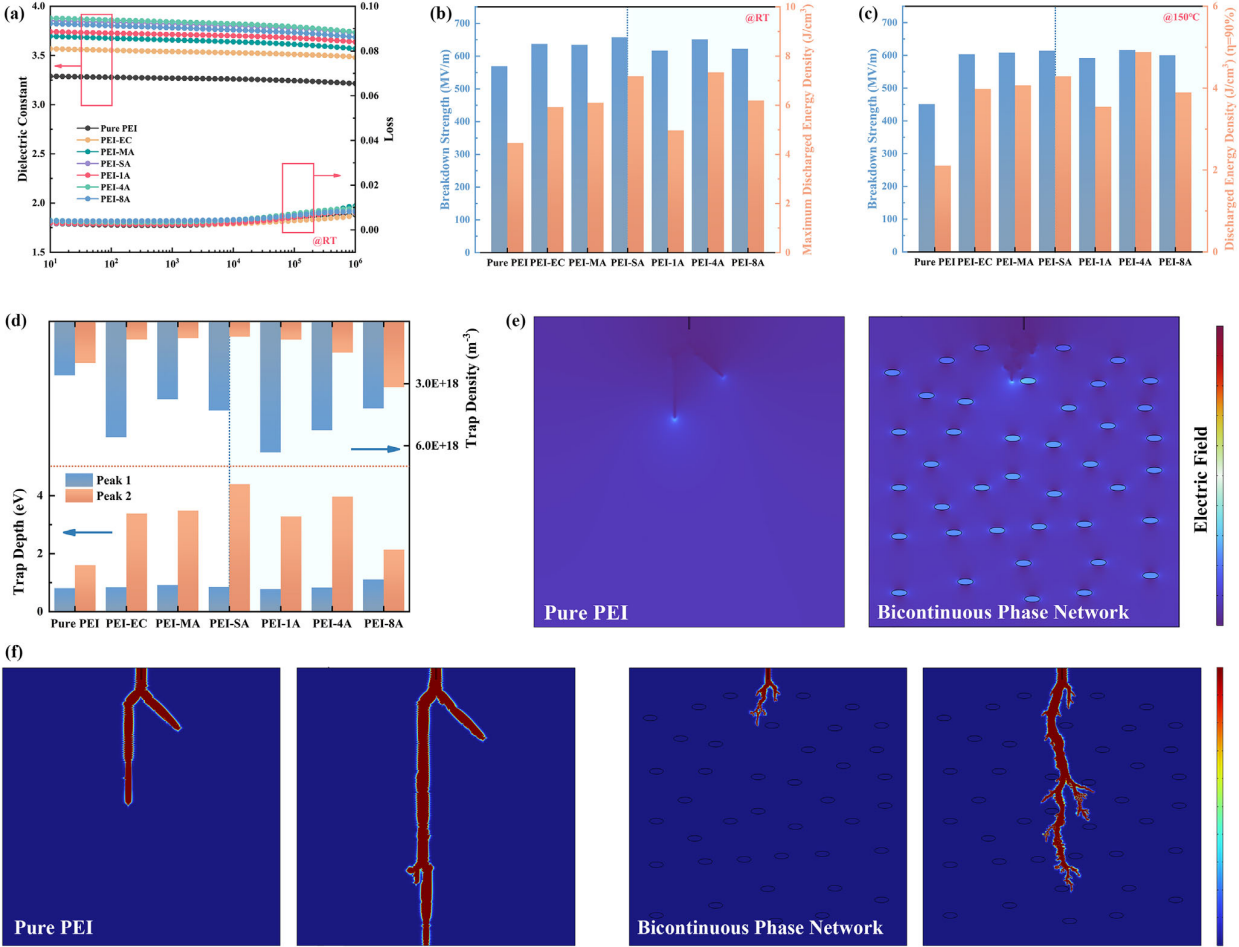
a) Frequency dependence of dielectric constant and loss for samples. Breakdown strength and discharged energy density of samples at b) RT and c) 150 °C. d) Trap depth and density from TSDC spectra of samples. e) Electric field distribution and f) electrical trees evolution of pure PEI and composites with the formed bicontinuous phase network.

Dielectric spectra of PEI and the composites are shown in **Figure** **4**a and Figure S13 (Supporting Information). It can be observed that the ε_*r*_ for PEI‐SA and PEI‐4A are 3.82 and 3.84, respectively, which are markedly enhanced compared to the value of 3.27 for pristine PEI at room temperature. The increased dielectric constant can be attributed to the improved mobility of dipoles, which is due to the increased interchain spacing, the increased density of dipoles resulting from the incorporation of LMs, and the induced interfacial polarization caused by the formation of bicontinuous phase network.^[^ ^44^ ^]^ With a further increasing content of LMs, a decrease in ε_*r*_ can be attributed to the lowering of interchain spacing, as well as interface polarization caused by restricted anti‐plasticization. Additionally, the composites with an optimal content of LMs represent stable dielectric performance across wide temperature range of 25–200 °C and frequency spectrum of 10^1^–10^6^ Hz (Figure S14, Supporting Information). The improvement of ε_*r*_ is attributed to the formation of a bicontinuous phase network and the introduction of interfacial polarization. For composites with higher content of LMs, the significant increase in dielectric loss is mainly caused by the decreased *T*_g_ and enhanced ionic conduction. Moreover, the polar groups on polymer chains in amorphous regions can also respond to the electric field, thus increase interchain spacing to enhance their mobility, resulting in augmented dipolar polarization. The breakdown strength *E*_b_ at room temperature and 150 °C are obtained by two‐parameter Weibull statistical analysis, and the values are presented in Figure 4b,c (see curves in Figures S15 and S16, Supporting Information). Composites with optimum content of LMs achieve a significantly improved *E*_b_, such as 1 wt.% PEI‐SA and 3 wt.% PEI‐4A. At room temperature, the *E*_b_ value of PEI‐SA and PEI‐4A can reach 658 MV m^−1^ and 651 MV m^−1^, respectively, much enhanced compared to that of 569 MV m^−1^ for pristine PEI. At 150 °C, PEI‐SA and PEI‐4A can maintain the value of 614 MV m^−1^ and 616 MV m^−1^ while the *E*_b_ of pristine PEI reduces to 452 MV m^−1^. Furthermore, the improved shape parameter *β* indicates superior compatibility between LMs and PEI. As the content of LMs increases, the degradation of *E*_b_ can be attributed to the dominant effects of plasticization and agglomeration of LMs. The anti‐plasticization enables the enhanced Young's modulus resulting from the maintained stiffness to achieve an improvement in *E*_b_, and the PEI‐rich phase acts as an insulating barrier to impede the spread of electric trees. The reduction in leakage current is also demonstrated to effectively result in improved *E*_b_ at 150 °C (see the outcomes in Figure S17, Supporting Information). It is evident that the leakage current density is significantly suppressed from 2.51 × 10^−7^ A cm^−2^ for PEI to 1.19 × 10^−7^ A cm^−2^ for PEI‐SA and 1.18 × 10^−7^ A cm^−2^ for PEI‐4A at 250 MV m^−1^ and 150 °C, signifying a decrease in conduction loss and an increase in efficiency.

The thermally stimulated depolarization current (TSDC) were conducted, and the typical curves of which featured two fitted peaks (Figure S18–S23, Supporting Information). The trap depth and density values are summarized in Figure 4d (see details in Tables S4 and S5, Supporting Information). The peak 1 plotted in blue is attributed to the release of trapped charge carriers, and the peak 2 plotted in red is associated with the glass transition.^[^ ^45^ ^]^ The larger depth and density of peak 1, e.g. 0.853 eV and 4.29 × 10^18^ m^3^ for PEI‐SA, 0.827 eV and 5.25 × 10^18^ m^3^ for PEI‐4A versus 0.810 eV and 2.60 × 10^18^ m^3^ for pristine PEI, enable the composites to hinder the detrapping and migration of injected charges. Moreover, the larger depth and lower density of peak 2 is also beneficial in suppressing the intense conduction caused by the glass transition (e.g., 3.966 eV and 1.50 × 10^18^ m^3^ for PEI‐4A versus 1.605 eV and 2.00 × 10^18^ m^3^ for pristine PEI). The UV‐visible spectroscopy and corresponding calculated bandgap of the composites (Figure S24 and Table S6, Supporting Information) represent small change and indicate that the addition of LMs does not degrade *E*_b_ by affecting the intrinsic breakdown field inside the matrix. The photoluminescence (PL) spectroscopy analysis is utilized to verify the information of charge traps. As demonstrated in Figure S25 (Supporting Information), a diminished luminescence intensity is evident upon incorporating LMs in comparison to pure PEI, suggesting that the majority of photoexcited carriers are captured and that the formation of additional charge traps occurs.^[^ ^46^ ^]^ Moreover, an increase in full width at half maximum can be indicative of enhanced trap density. Moreover, to provide additional clarification regarding the trap chemistry, the electron affinity (EA) and ionization energy (IE) of the selected LMs are calculated via DFT calculations. Normally, the smaller IE indicates that the molecule in its positively charged form is more stable and features a deeper trap for holes. Conversely, a larger EA features a deeper trap for electrons.^[^ ^47^ ^]^ The calculations are presented in Table S7 (Supporting Information). The lower IE of SA and 4A indicates the formation of deeper traps for holes. Moreover, the larger EA for LMs can also present the capability to capture more electrons. The deep charge traps induced by the introduction of LMs with lower IE and higher EA can be capable of capturing electric charge carriers such as electrons and holes. Moreover, as demonstrated in Figure 1b and the calculated total density of states spectra (TDOS) in Figure S26 (Supporting Information), due to the lower HOMO levels and higher LUMO levels of LMs, they can also act as the anti‐traps to scatter the charge carriers and impede their transportation.

As shown in Figure 4e,f, the phase field method is also utilized to simulate the initiation and real‐time evolution of the electrical treeing process, as well as the electric field distribution in both pure PEI and composites with the formed bicontinuous phase network. It can be seen that the PEI‐rich phase bears a higher electric field, thereby serving as an insulation phase, as mentioned above. During the breakdown process, all the paths nucleate from the top electrode and exhibit random growth patterns. In pure PEI, the electrical dendrites demonstrate a rapid and straight growth pattern with increasing applied voltage. However, a slower growth for electrical dendrites can be seen in composites. Besides, the PEI droplets can also force the breakdown paths to propagate along the interface, leading to more circuitous and protracted pathways. The extension of these paths along the in‐plane direction can disperse the overall breakdown path and dissipate the electrostatic energy that drives the propagation of breakdown paths.^[^ ^24^ ^]^ Briefly, the establishment of a bicontinuous phase network in composites is beneficial for the improvement of breakdown strength due to the lower speed of breakdown process.

The discharged energy densities and efficiencies of the composites at room temperature and 150 °C (Figures S27 and S28, Supporting Information) are calculated from the corresponding electric displacement‐electric field (D‐E) loops (Figure S29–S40, Supporting Information). Owing to the improved ε_*r*_ and *E*_b_, the *U*_d_ values are remarkably augmented with the addition of optimal content of LMs. As shown in Figure 4b,c, the maximum *U*_d_ at room temperature reaches 7.18 J cm^−3^ for PEI‐SA and 7.34 J cm^−3^ for PEI‐4A, well over 4.46 J cm^−3^ of pristine PEI, while the *U*_d_ with *η* of 90% at 150 °C achieves 4.29 J cm^−3^ for PEI‐SA and 4.88 J cm^−3^ for PEI‐4A, highly surpassing the value of 2.11 J cm^−3^ for pristine PEI. In a word, the heightened polarizability, suitable solubility, appropriate alkyl chain length, and adequate phase separation morphology all synergistically contribute to raising *U*_d_ by anti‐plasticization. Usually, phase separation only can be observed in crystalline polymers.^[^ ^48^, ^49^, ^50^ ^]^ But we obtained a similar structure in liner polymers by the introduction of LMs. According to a simple doping strategy, the composite effectively decouples the inverse relationship between *E*_b_ and ε_*r*_ in polymer dielectrics by anti‐plasticization, offering a promising approach through phase structure modulation for the fabrication of dielectric film suitable for advanced high‐temperature energy application.

**Figure 5:**
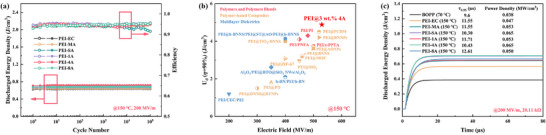
a) Cycling stability of samples at 150 °C and 200 MV m^−1^. b) Comparison of the discharged energy density above 90% efficiency of this work and other PEI‐based composites at 150 °C. c) Fast discharge capability of PEI‐LMs composites.

Cyclic charging‐discharging tests were performed at different temperatures and electric field to evaluate the reliability of the composites, as illustrated in **Figure** **5**a and Figure S41 (Supporting Information). All the samples exhibit stable energy storage performance over 10^5^ cycles, with PEI‐4A maintaining superior *U*_d_ and *η* under the electric field as high as 400 MV m^−1^ at 150 °C. Moreover, a stable cyclic performance is also achieved at different temperatures such as room temperature, 60 and 100 °C. To facilitate a comprehensive comparative evaluation of the energy storage performance in this work and other literatures, the *U*_d_ with *η* of 90% at 150 °C of various polymer dielectrics were summarized, as shown in Figure 5b.^[^ ^51^, ^52^, ^53^, ^54^, ^55^, ^56^, ^57^, ^58^, ^59^, ^60^, ^61^, ^62^, ^63^, ^64^, ^65^, ^66^, ^67^ ^]^ It is evident that the PEI‐4A composites represent a notably high *U*_d_ among the reported values obtained through strategies of blending, doping, molecular design, and multilayer structure design. Moreover, fast discharge tests of the composites (150 °C) and commercial BOPP (70 °C) were comparatively carried out (see details in Figure 5c). PEI‐SA and PEI‐4A achieve significantly higher power density of 0.065 MW cm^−3^ compared to that of 0.038 MW cm^−3^ for BOPP. In addition, the relatively shorter discharged time (τ_0.95_, defined as the time required to discharge 95% of the charged energy) of 10.30 µs and 10.43 µs for PEI‐SA and PEI‐4A, respectively, indicates a substantially faster release of electrical energy, signifying potential for large power density applications.

## 3 Conclusion

The effect of anti‐plasticization on the dielectric properties of polymers has been investigated by successfully preparing PEI composites incorporating different types of plasticizers through a simple solution‐casting process. A bicontinuous phase network was formed inside the polymer, where the PEI‐rich phase acts as an insulation phase and LMs‐rich phase serves as a dielectric phase to simultaneously enhance the *E*_b_ and ε_*r*_ through the electric field redistribution and interface polarization. The adequate synergistic effect of polar and non‐polar groups for 4A provides high interaction and compatibility with the PEI matrix, which is beneficial to preserve thermal and mechanical properties of the composites. Therefore, the improved ε_*r*_ of 3.75 as well as *E*_b_ of 616 MV m^−1^ were achieved in PEI‐4A and a relatively high *U*_d_ of 4.88 J cm^−3^ with *η* of 90% at 150 °C was obtained. Moreover, the composites exhibit remarkable stability in capacity, maintaining performance over 10^5^ charging‐discharging cycles at 150 °C and 200 MV m^−1^. This work introduces synthesis of novel all‐organic composites composed of bicontinuous phase network by integrating plasticizers. The composite effectively decouples the inverse relationship between *E*_b_ and ε_*r*_ in polymer dielectrics by anti‐plasticization, offering a promising approach through phase structure modulation for the fabrication of dielectric film suitable for advanced high‐temperature energy application.

## Conflict of Interest

The authors declare no conflict of interest.

## Supporting information



Supporting Information

## Data Availability

The data that support the findings of this study are available from the corresponding author upon reasonable request.

## References

[1] H. Li , Y. Zhou , Y. Liu , L. Li , Y. Liu , Q. Wang , Chem. Soc. Rev. 2021, 50, 6369. 34100032 10.1039/d0cs00765j

[2] M. Yang , F. Yuan , W. Shi , W. Ren , M. Guo , C. Ouyang , L. Zhou , N. Sun , Y. Xiao , E. Xu , X. Zhang , Y. Wei , X. Deng , C. Nan , X. Wang , Y. Shen , Adv. Funct. Mater. 2023, 33, 2214100.

[3] X. Li , P. Hu , J. Jiang , J. Pan , C.‐W. Nan , Y. Shen , Adv. Mater. 2025, 37, 2411507. 10.1002/adma.202411507 39846312

[4] M. Yang , W. Ren , M. Guo , Y. Shen , Small 2022, 18, 2205247,. 10.1002/smll.202205247 36266932

[5] J. Liu , L. Ji , J. Yu , S. Ding , S. Luo , B. Chu , J. Xu , R. Sun , S. Yu , Chem. Eng. J. 2023, 456, 140950.

[6] J. Hao , S. Shukla , R. Gurnani , M. Mukherjee , H. Sahu , A. Khomane , P. Aklujkar , M. Desai , C. Wu , R. Ramprasad , G. Sotzing , Y. Cao , Adv. Mater. 2025, 37, 2417625. 10.1002/adma.202417625 40026039

[7] X. Zhao , L. Zhang , F. Zhenhao , Y. Huang , Y. Hu , M. Shen , Z. Wang , Y. He , D. Wang , Q. Zhang , Nat. Commun. 2025, 16, 5570. 40593587 10.1038/s41467-025-60683-8 PMC12215717

[8] Z. Li , Y. Zhang , Z. Pan , X. Fan , P. Li , W. Chen , J. Liu , W. Li , ACS Appl. Mater. Interfaces 2024, 16, 10756. 38367030 10.1021/acsami.3c18733

[9] M. Yang , H. Li , J. Wang , W. Shi , Q. Zhang , H. Xing , W. Ren , B. Sun , M. Guo , E. Xu , N. Sun , L. Zhou , Y. Xiao , M. Zhang , Z. Li , J. Pan , J. Jiang , Z. Shen , X. Li , L. Gu , C.‐W. Nan , X. Wang , Y. Shen , Nat. Energy 2024, 9, 143.

[10] S. Huang , K. Liu , W. Zhang , B. Xie , Z. Dou , Z. Yan , H. Tan , C. Samart , S. Kongparakul , N. Takesue , H. Zhang , Polym. Rev. 2023, 63, 515.

[11] J.‐W. Zha , Y. Tian , M.‐S. Zheng , B. Wan , X. Yang , G. Chen , Mater. Today Energy 2023, 31, 101217.

[12] Z. Zhang , D. H. Wang , M. H. Litt , L. S. Tan , L. Zhu , Angew. Chem. 2018, 130, 1544. 10.1002/anie.201710474 29266592

[13] C. Yuan , Y. Zhou , Y. Zhu , J. Liang , S. Wang , S. Peng , Y. Li , S. Cheng , M. Yang , J. Hu , B. Zhang , R. Zeng , J. He , Q. Li , Nat. Commun. 2020, 11, 3919. 32764558 10.1038/s41467-020-17760-x PMC7411043

[14] K. Fan , X. Li , X. Liu , X. He , Z.‐M. Dang , Adv. Mater. 2025, 37, 2417181. 10.1002/adma.202417181 40150945

[15] M. Yang , L. Zhou , X. Li , W. Ren , Y. Shen , Adv. Mater. 2023, 35, 2302392. 10.1002/adma.202302392 37196180

[16] V. Marturano , P. Cerruti , V. Ambrogi , Phys. Sci. Rev. 2017, 2, 20160130.

[17] A. D. Godwin , in Applied Plastics Engineering Handbook, 3rd ed. (Ed: M. Kutz ), William Andrew Publishing, 2024, pp. 595.

[18] M. G. A. Vieira , M. A. Da Silva , L. O. Dos Santos , M. M. Beppu , Eur. Polym. J. 2011, 47, 254.

[19] L. Mascia , Y. Kouparitsas , D. Nocita , X. Bao , Polymers 2020, 12, 769. 32244603 10.3390/polym12040769 PMC7240542

[20] A. Marcilla , M. BeltrÁN , in Handbook of Plasticizers, 3rd ed. (Ed: G. Wypych ), ChemTec Publishing, 2017, pp. 119.

[21] J.‐W. Zha , M. Xiao , B. Wan , X. Wang , Z.‐M. Dang , G. Chen , Prog. Mater. Sci. 2023, 140, 101208.

[22] H. Yuan , Y. Zhou , Y. Zhu , S. Hu , C. Yuan , W. Song , Q. Shao , Q. Zhang , J. Hu , Q. Li , H. Jinliang , J. Phys. D: Appl. Phys. 2020, 53, 475301.

[23] Y. Chernyak , J. Chem. Eng. Data 2006, 51, 416.

[24] Z. Tong , Y. Xie , Y. Zhang , J. Mol. Liq. 2018, 259, 65.

[25] K. A. Gebru , C. Das , Chin. J. Chem. Eng. 2017, 25, 911.

[26] A. Bonifacio , L. Bonetti , E. Piantanida , L. De Nardo , Eur. Polym. J. 2023, 197, 112360.

[27] H.‐L. Chen , J.‐W. You , R. S. Porter , J. Polym. Res. 1996, 3, 151.

[28] B. Silverman , Macromolecules 1989, 22, 3768.

[29] S. Devasahayam , D. J. Hill , J. W. Connell , J. Appl. Polym. Sci. 2006, 101, 1575.

[30] G. Xiaohong , C. Q. Yang , Text. Res. J. 2000, 70, 64.

[31] G. Goyal , M. Garg , J. Quamara , Radiat. Eff. Defects Solids 2008, 163, 131.

[32] M. C. Staub , C. Y. Li , Polym. Crystallization 2018, 1, 10045.

[33] G. Liu , Y. Feng , T. Zhang , C. Zhang , Q. Chi , Y. Zhang , Y. Zhang , Q. Lei , J. Mater. Chem. A 2021, 9, 16384.

[34] L. Cheng , H. Gao , K. Liu , H. Tan , P. Fan , Y. Liu , Y. Hu , Z. Yan , H. Zhang , Macromol. Mater. Eng. 2022, 307, 2100822.

[35] A. Garcia , M. Iriarte , C. Uriarte , J. Iruin , A. Etxeberria , J. Del Rio , Polymer 2004, 45, 2949.

[36] D. K. Chattopadhyay , D. C. Webster , Prog. Polym. Sci. 2009, 34, 1068.

[37] P. Bergquist , Y. Zhu , A. A. Jones , P. T. Inglefield , Macromolecules 1999, 32, 7925.

[38] G.‐H. Kim , D. Lee , A. Shanker , L. Shao , M. S. Kwon , D. Gidley , J. Kim , K. P. Pipe , Nat. Mater. 2015, 14, 295. 25419813 10.1038/nmat4141

[39] X. Huang , P. Jiang , T. Tanaka , IEEE Electr. Insul. Mag. 2011, 27, 8.

[40] K. Shirasu , A. Nakamura , G. Yamamoto , T. Ogasawara , Y. Shimamura , Y. Inoue , T. Hashida , Compos. Part Appl. Sci. Manuf. 2017, 95, 152.

[41] T. Venkategowda , L. H. Manjunatha , P. R. Anilkumar , Mater. Today: Proc. 2022, 54, 395.

[42] T. Rath , S. Kumar , R. Mahaling , M. Mukherjee , C. Das , K. Pandey , A. Saxena , Polym. Compos. 2006, 27, 533.

[43] G. Raju , C. Rajeswari , R. Balannavar , K. Kodancha , IJEST 2018, 10, 30.

[44] M. Z. Yang , W. B. Ren , M. F. Guo , Y. Shen , Small 2022, 18, 2205247. 10.1002/smll.202205247 36266932

[45] T. Zeng , Q. Li , D. Liu , J. Fu , L. Zhong , J. He , Q. Li , C. Yuan , Mater. Horiz. 2024, 11, 1539. 38251735 10.1039/d3mh01928d

[46] Y. Huang , K. Wu , M. Bell , A. Oakes , T. Ratcliff , N. A. Lanzillo , C. Breneman , B. C. Benicewicz , L. S. Schadler , J. Appl. Phys. 2016, 120, 055102.

[47] H. Zhang , Y. Shang , X. Wang , H. Zhao , B. Han , Z. Li , J. Mol. Model. 2013, 19, 5429. 24193211 10.1007/s00894-013-2028-0

[48] J. Chen , T. Li , Z. Lv , Y. Zhai , W. Liao , Q. Zhang , Appl. Phys. Lett. 2025, 127, 012903.

[49] S. Zhao , L. Zhou , J. Zhang , Y. Shen , C.‐W. Nan , Giant 2024, 20, 100340.

[50] W. Zhu , G. Rui , W. Lu , E. L. Briggs , J. Bernholc , Q. M. Zhang , Nano Energy 2024, 128, 109898.

[51] J. Ding , W. Xu , X. Zhu , Z. Liu , Y. Zhang , Z. Jiang , Nano Res. 2023, 16, 10183.

[52] J. Yan , H. Wang , J. Zeng , X. Zhang , C. W. Nan , S. Zhang , Small 2023, 19, 2304310. 10.1002/smll.202304310 37340581

[53] X. Li , H. Luo , C. Yang , F. Wang , X. Jiang , R. Guo , D. Zhang , ACS Appl. Mater. Interfaces 2023, 15, 41828. 37632445 10.1021/acsami.3c06778

[54] Z. Liu , T. Wang , L. Zhu , Z. Jiang , Y. Zhang , Compos. Part Appl. Sci. Manuf. 2023, 175, 107829.

[55] J. Li , X. Liu , B. Huang , D. Chen , Z. Chen , Y. Li , Y. Feng , J. Yin , H. Yi , T. Li , Mater. Horiz. 2023, 10, 3651. 37340861 10.1039/d3mh00499f

[56] S. Cheng , M. Yang , J. Fu , R. Wang , J. He , Q. Li , IET Nanodielectr. 2023, 6, 237.

[57] T. Zhang , L. Yang , J. Ruan , C. Zhang , Q. Chi , Macromol. Mater. Eng. 2021, 306, 2100514.

[58] C. Yuan , Y. Zhou , Y. Zhu , J. Liang , S. Wang , S. Peng , Y. Li , S. Cheng , M. Yang , J. Hu , Nat. Commun. 2020, 11, 3919. 32764558 10.1038/s41467-020-17760-x PMC7411043

[59] M. Fan , P. Hu , Z. Dan , J. Jiang , B. Sun , Y. Shen , J. Mater. Chem. A 2020, 8, 24536.

[60] C. Yan , H. Luo , X. Liu , Y. Liu , S. Chen , Mater. Today Sustain. 2023, 21, 100310.

[61] C. Zhou , W. Xu , Y. Zhang , C. Yu , X. Liu , Z. Jiang , C. Zhang , Y. Shang , H. Zhang , ACS Appl. Mater. Interfaces 2023, 15, 8471. 36725214 10.1021/acsami.2c20558

[62] X. Ding , Z. Pan , Y. Zhang , S. Shi , Y. Cheng , H. Chen , Z. Li , X. Fan , J. Liu , J. Yu , Adv. Mater. Interfaces 2022, 9, 2201100.

[63] J. Yan , J. Wang , J. Zeng , Z. Shen , B. Li , X. Zhang , S. Zhang , J. Mater. Chem. C 2022, 10, 13157.

[64] H. Chen , Z. Pan , Y. Cheng , X. Ding , J. Liu , Q. Chi , M. Yang , J. Yu , Z.‐M. Dang , J. Mater. Chem. A 2022, 10, 1579.

[65] H. Li , L. Ren , D. Ai , Z. Han , Y. Liu , B. Yao , Q. Wang , InfoMat 2020, 2, 389.

[66] Y. Wang , M. Feng , Q. Chi , in 2022 IEEE Int. Conf. on High Voltage Engineering and Applications (ICHVE) , Chongqing, China, September 2022.

[67] A. Azizi , M. R. Gadinski , Q. Li , M. A. AlSaud , J. Wang , Y. Wang , B. Wang , F. Liu , L. Q. Chen , N. Alem , Adv. Mater. 2017, 29, 1701864. 10.1002/adma.201701864 28714119

