# Supplementary material for: Bicontinuous Phase Network Formed by Anti‐Plasticization Enhances Energy Storage Performance in Polyetherimide Dielectric Film
Source: Adv Sci (Weinh). 2025 Sep 15;12(45):e12343. doi: 10.1002/advs.202512343 (PMC12677604; doi:10.1002/advs.202512343)
Supplement: Supplementary file 1 — Supporting Information [file ADVS-12-e12343-s001.docx]

Supporting Information

Bicontinuous Phase Network formed by Anti-plasticization enhances Energy Storage Performance in Polyetherimide Dielectric Film

Xin Li, Le Zhou, Yao Xiao, Erxiang Xu, Taoyuan Yu, Mufeng Zhang, Minzheng Yang, Weibin Ren, Penghao Hu*, Yang Shen*

Experimental Section

*Materials*: Polyetherimide pellets (PEI, Ultem 1000) were purchased from General Electric (GE). Succinic anhydride (SA) was obtained from Beijing Honghu Lianhe Chemical Products Co., Ltd, and Ethylene carbonate (EC) was purchased from Boer. Maleic anhydride (MA) was supplied by Titan, while Methylsuccinic anhydride (1A) was provided by Bidepharm. Butylsuccinic anhydride (4A) and n-Octylsuccinic anhydride (8A) were obtained from Meryer and Macklin, respectively. All chemicals were stored in a dry environment and used as received without further purification. N-methylpyrrolidone (NMP) was supplied by China National Chemicals Corporation Ltd.

*Preparation of PEI-LMs composites*: Before the preparation, all materials were kept into a vacuum oven at 60 °C for 30 min to remove the water. The PEI pellets and a certain amount of LMs were dissolved in NMP with a concentration of 150 mg mL^-1^ through vigorous stirring at 60 °C for 4 h to obtain the uniform dispersion solution. Then, the as-prepared solution was cast onto a clean glass slide and moved into the vacuum oven at 40 ℃ for 12 h, and 200 °C for 3 h to remove the residual solvent. Finally, the films were peeled form substrates and were dried at 110 ℃ for 2 h to evaporate residual water. The thickness of the obtained films is about 15 µm.

*Characterization*: The surface and cross-sectional micromorphology were investigated by a scanning electron microscope (SEM, ZEISS MWRLIN compact). X-rays diffraction (XRD) patterns were measured by a Rigaku D/max-2550. Fourier transform infrared spectroscopy (FTIR) analysis was performed on a Nicolet AVATAR 360ESP FTIR spectrometer. Raman spectroscopy (RS) was performed using a high-resolution Raman spectrometer (HORIBA HR800) with the 532 nm laser source. AFM-IR measurements were implemented using a NanoIR3 (Bruker) and the polarized IR laser was set at 920 and 1166 cm^-1^ during the scanning. Differential scanning calorimetry (DSC) was employed with the heating rate of 10 ℃ min^-1^ in nitrogen atmosphere by using a DSC Q2000 device (TA Instrument). Thermal gravimetric analysis (TGA) was performed with TA Q5000IR device (TA Instrument) over the temperature range from 25 – 500 ℃ at a heating rate of 5 ℃/min in nitrogen atmosphere. The dynamic mechanical analysis (DMA, TA Q800), the thermal mechanical analysis (TMA, TA Q400 EM), and tensile testing (SHIMADZU AGS-X, Japan) were conducted for film samples 0.05 – 0.07 mm thick, 25 mm wide and 50 – 60 mm long. DMA analysis was performed at a fixed frequency of 1 Hz from 25 ℃ to 250 ℃ at a heating rate of 2 ℃/min. TMA analysis was carried out at a heating rate of 10 ℃/min with an inserted force of 50 mN. Tensile stress tests were conducted at room temperature with a 20 mm $\text{min}\text{-1}$ tensile speed. Nano-indenter (Keysight Technologies G200) was performed to measure the Young’s moduli.

Copper electrodes were sputtered on both sides of the prepared composites and the diameters are 3 mm and 15 mm to measure the dielectric properties under high electric field (*D-E* loop, breakdown strength, and leakage current) and low electric field (dielectric spectra, thermally stimulated depolarization current and fast charge-discharge energy density), respectively. Dielectric spectra and loss were tested by Novocontrol broadband dielectric/impedance spectrometer (Novocontrol Technologies GmbH& Co. KG.) with a liquid nitrogen cooling system in the frequency range of 10 – 10^6^ Hz and temperature range of 25-200 °C at 1 Vrms. Thermally stimulated discharge currents (TSDC, Novocontrol) were obtained for the TSDC study. A DC electric field of 40 MV m^-1^ was applied to polarize the samples at 180 ℃ for 10 min. Then the samples were rapidly cooled down to -100 ℃ at a rate of 10 ℃ $\text{min}\text{-1}$ while the electric field was maintained. After the polarization, the electric field was eliminated and the samples were heated to 220 ℃ at a rate of 3 ℃ min^-1^. The depolarization current corresponding to the temperature was recorded concomitantly. Electric displacement - electric field (*D-E*) loops were measured using a polarization loop & dielectric breakdown test system (PolyK Technologies, LLC) at 100 Hz during which samples are in temperature resistant silicone oil to control the temperature. Cyclic charge–discharge tests were performed using the same system. Breakdown strength was tested using a Trek 20/20B instrument at a voltage ramp of 500 V s^-1^, and a two-parameter Weibull distribution equation P(*E*)=1-exp[-(*E*/*E*_b_)^β^] was employed to determine the breakdown strength, where P(*E*) was the cumulative probability of dielectric failure and *E* was the measurement breakdown data. The leakage currents with respect to the electric field were tested using a ferroelectric test system (Premier II, Radiant Technologies, Inc.). Fast charge-discharge tests were conducted using a PK-DIS20012 system (PolyK Technologies, LLC), with a given field *E* of 200 MV m^-1^ and a load resistor of 20.11 kΩ.

The trap depth and density values can be calculated according to the half-width method by TSDC curves [1].

$$\text{Q}_{\text{TSDC}}\text{=}\frac{\text{60}}{\text{β}}\int_{\text{T}_{\text{0}}}^{\text{T}_{\text{1}}} \text{I(T)}\text{dT}$$

$$\text{E=}\frac{\text{2.47}\text{T}_{\text{m}}^{\text{2}}\text{k}}{\text{∆T}}$$

where, $\text{β}$ is the heating rate, $\text{T}_{\text{0}}$ and $\text{T}_{\text{1}}$ represent the starting and ending temperatures of a peak, respectively. $\text{I(T)}$ describes the TSDC curves. $\text{T}_{\text{m}}$ is the temperature corresponding to the peak position, and $\text{∆T}$ is the difference in temperature corresponding to the half peak. $\text{k}$ is the Boltzmann constant.

*Density functional theory (DFT) calculation*: The geometrical structures of polyetherimides and plasticizers were optimized and analyzed with B3LYP/6-31G(d,p) level using Gaussian 09 soft programs and the Gauss-view molecular visualization package. The electrostatic potential distribution and a few quantum chemical parameters such as energy gap (HOMO-LUMO) and dipole moment were calculated [2]. Multiwfn and VMD software were used to analyze and visualized the optimized structure [3, 4].

*Molecular dynamics simulation*: Molecular dynamics (MD) simulation had been performed to elucidate macroscopic properties through molecular interactions. We placed the molecules of each compound in cubic units on the 50 Å side to obtain the initial system. The position and direction are used as quasi random numbers [5]. The internal geometric shape of each molecule is arranged according to the optimized structure as described above. Using the steepest descent method to minimize the energy of the system. Then, perform a 400 ps MD calculation at 300 K and atmospheric pressure, followed by a 200 ps equilibrium calculation. We conducted sampling simulations on NPT components for over 10ns. The short-range cutoff value for Vander Waals and Coulomb interactions in direct space is 10 Å. The optimal structures are achieved by the energy minimized method. After equilibrium, the last few hundred picoseconds of the trajectory files were used to calculate the Hildebrand solubility parameters and it was expressed as the square root of the cohesive energy density (CED) [6].

*Phase-field simulation*: To further investigate the effect of formed bicontinuous phase network on the breakdown strength for composites, a phase-field model was used to simulate the breakdown process. A scalar phase field variable $\text{η}\text{(}\text{r}\text{,t}\text{)}$ was introduced to characterize the breakdown behavior, where $\text{η}\text{(}\text{r}\text{,t}\text{)}$=1 represents the breakdown region, $\text{η}\text{(}\text{r}\text{,t}\text{)}$=0 represents the non-breakdown region, and the transitional region represents the interface region. And $\text{η}\text{(}\text{r}\text{,t}\text{)}$ is both spatially and temporally dependent. The breakdown region in the phase-field model will automatically grow or not, depending on the kinetic equation. In an inhomogeneous system, the free energy considering synergistic contributions from the phase separation, the interface, and the electric field can be written by

$$\text{F=}\int_{\text{V}} \text{[}\text{f}_{\text{sep}}\text{(}\text{η}\text{(}\text{r}\text{))}\text{+}\frac{\text{1}}{\text{2}}\text{γ}\left| \text{∇}\text{(}\text{η}\text{(}\text{r}\text{)} \right|^{\text{2}}\text{+}\text{f}_{\text{elec}}\text{(}\text{r}\text{)}\text{d}\text{V}$$

where the first term denotes the phase separation energy, which can be delineated by a double-well function $\text{f}_{\text{sep}}\text{(}\text{η}\text{(}\text{r}\text{)}\text{)=}\text{α}\text{η}^{\text{2}}{\text{(1-}\text{η}\text{)}}^{\text{2}}$ with $\text{α}$ representing a positive coefficient defining the energy barrier of the phase separation. The second term is the gradient energy density with $\text{γ}$ representing the gradient energy coefficient in isotropic approximation. The final term represents the electrostatic energy density of the system and it can be expressed as

$$\text{f}_{\text{elec}}\text{(}\text{r}\text{)=}\frac{\text{1}}{\text{2}}\text{ε}_{\text{0}}\text{ε}_{\text{ij}}\text{(}\text{r}\text{)}\text{E}_{\text{i}}\text{(}\text{r}\text{)}\text{E}_{\text{j}}\text{(}\text{r}\text{)}$$

where $\text{ε}_{\text{ij}}\text{(}\text{r}\text{)}$ is the spatially dependent relative dielectric permittivity tensor, $\text{E}_{\text{j}}\text{(}\text{r}\text{)}$ is the total electric component. In our work, the simulation model is composed of LMs matrix and PEI droplet with a disk-like morphology. The major axis and minor axis of PEI droplet are set as 1.5 μm and 600 nm, respectively. The dielectric constant of PEI and LMs were set to 3.2 and 80, respectively.

The breakdown phase evolution is described by a modified Allen-Cahn equation:

$$\frac{\text{∂}\text{η}\text{(}\text{r}\text{,t)}}{\text{∂t}}\text{=-}\text{L}_{\text{0}}\text{H}\text{(}\text{f}_{\text{elec}}\text{-}\text{f}_{\text{critical}}\text{)[}\frac{\text{∂}\text{f}_{\text{sep}}\text{(η(}\text{r}\text{))}}{\text{∂}\text{η}\text{(}\text{r}\text{,t)}}\text{-}\text{γ}\text{∇}^{\text{2}}\text{η}\text{(}\text{r}\text{,t)+}\frac{\text{∂}\text{f}_{\text{elec}}\text{(}\text{r}\text{)}}{\text{∂}\text{η}\text{(}\text{r}\text{,t)}}\text{]}$$

where $\text{L}_{\text{0}}$ is the kinetic coefficient related to the interface mobility, $H(\text{f}_{\text{elec}}-\text{f}_{\text{critical}})$ is the Heaviside unit step function and it can be defined as $H\left( \text{f}_{\text{elec}}\text{<}\text{f}_{\text{critical}} \right)\text{=0}$ and $H\left( \text{f}_{\text{elec}}\text{>}\text{f}_{\text{critical}} \right)\text{=1}$. $\text{f}_{\text{critical}}$ is a position-dependent material con1` stant related to the maximal energy density of each constituent in the composite. The purpose of introducing the Heaviside function into the Allen-Cahn equation is to ensure that the breakdown phase can only grow if the electric energy of a local point is greater than its maximal energy endurance [7]. The phase-field simulations were performed utilizing the above equations with the COMSOL Multiphysics 6.2 software.


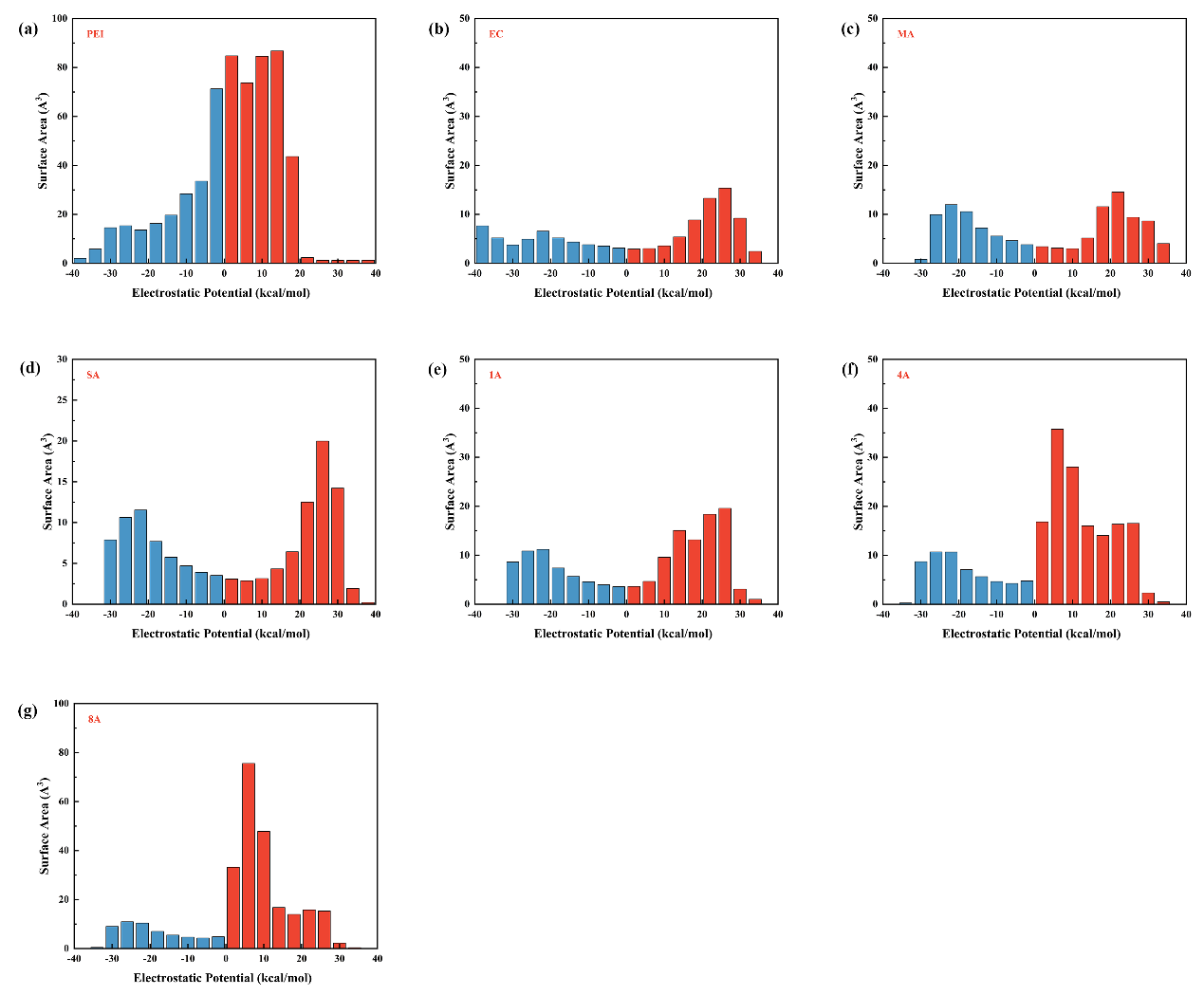


**Figure S1**. The electrostatic potential distribution and histogram of (a) PEI and (b) EC, (c) MA, (d) SA, (e) 1A, (f) 4A, (g) 8A.


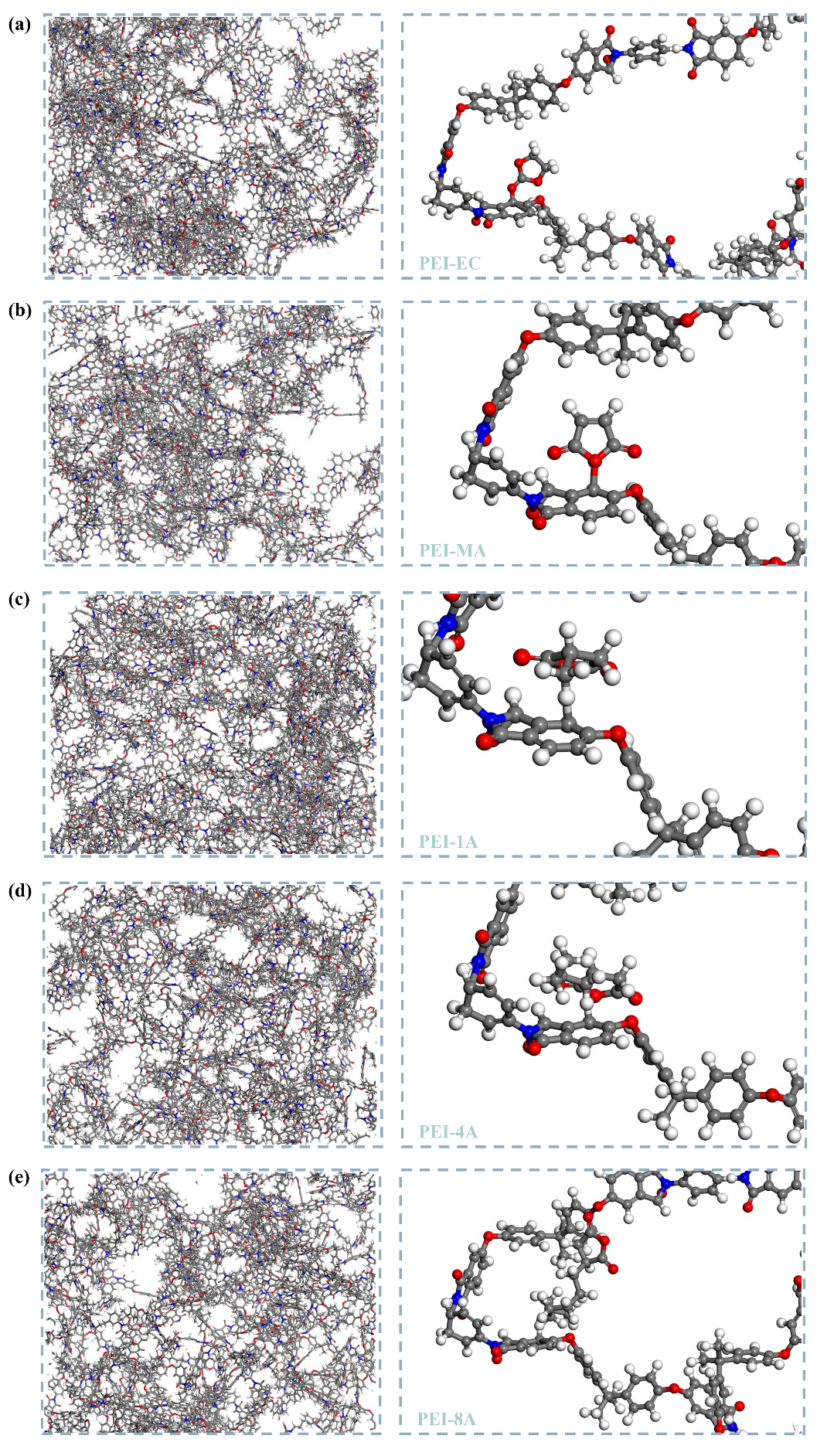


**Figure S2.** MD results of the interaction in composites: (a) PEI-EC, (b) PEI-MA, (c) PEI-1A, (d) PEI-4A, (e) PEI-8A.


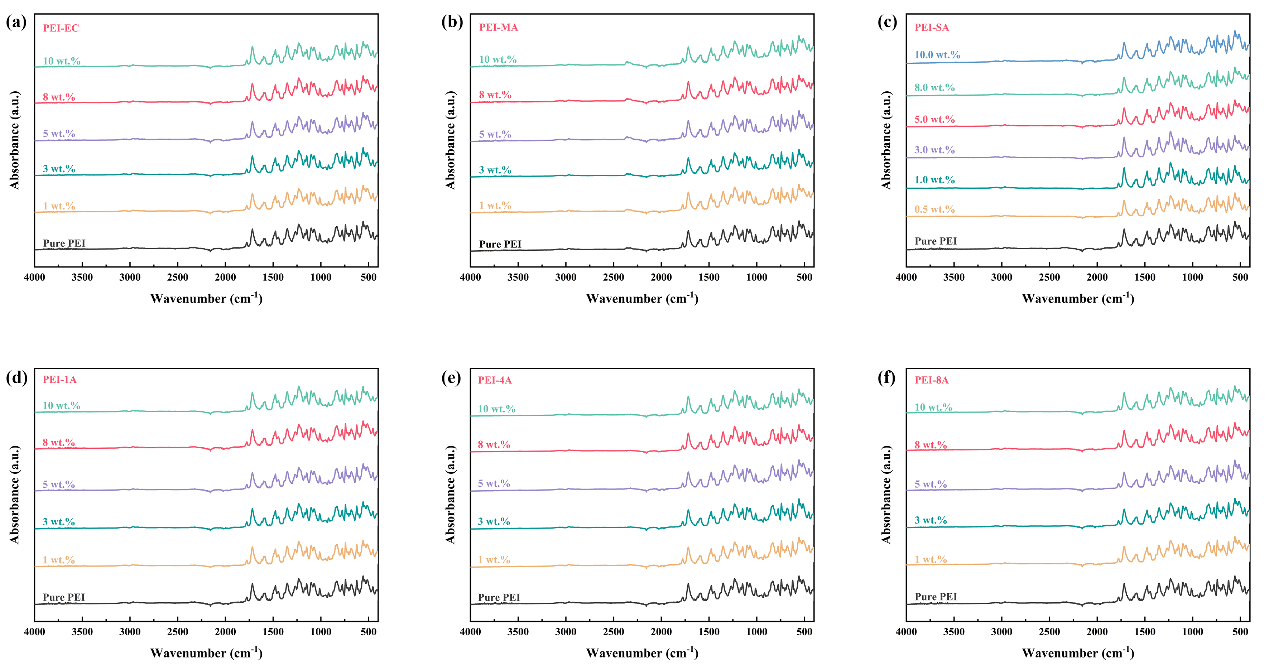


**Figure S3.** FTIR spectra of composites: (a) PEI-EC, (b) PEI-MA, (c) PEI-SA, (d) PEI-1A, (e) PEI-4A, (f) PEI-8A.


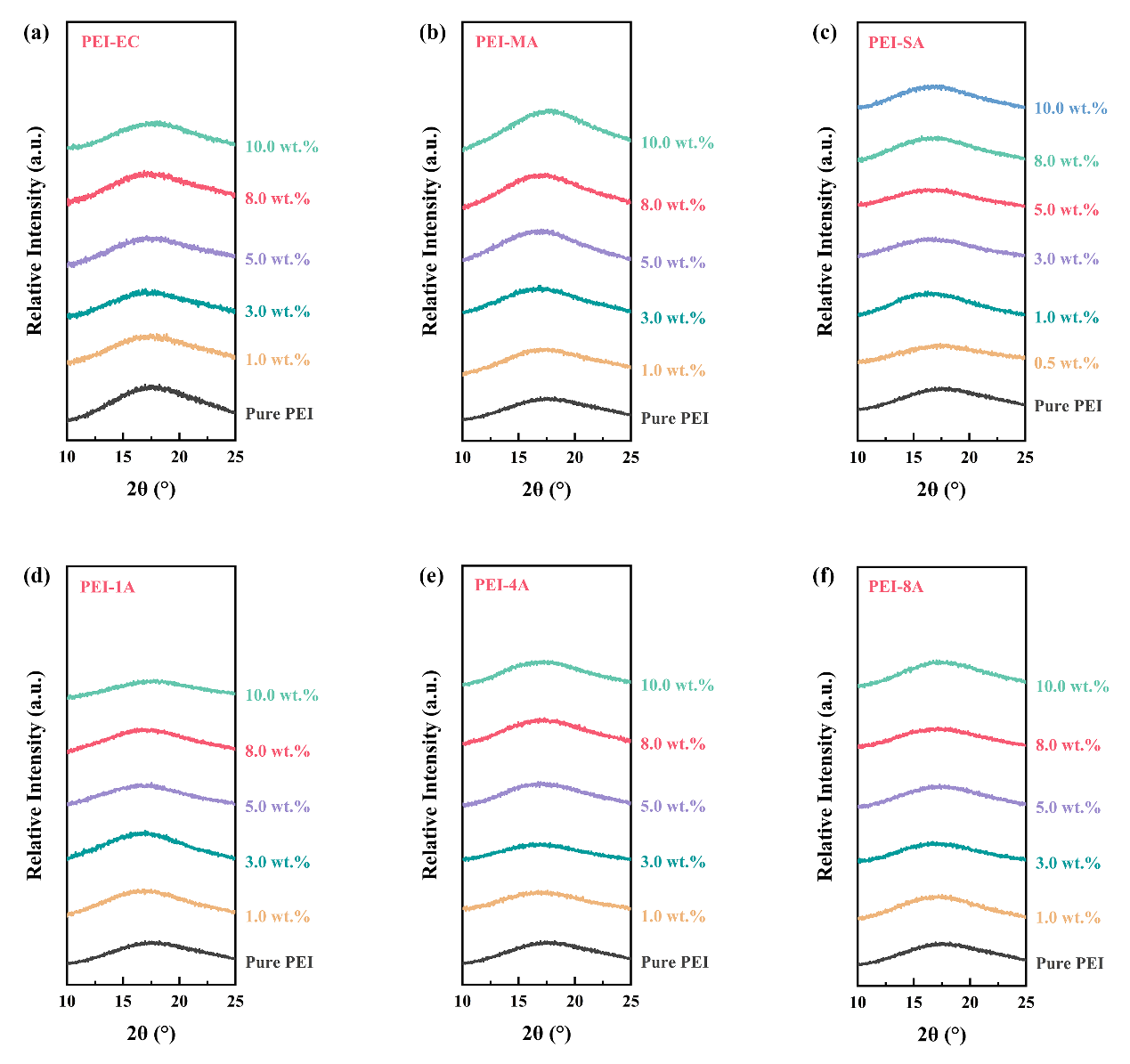


**Figure S4.** XRD data of composites: (a) PEI-EC, (b) PEI-MA, (c) PEI-SA, (d) PEI-1A, (e) PEI-4A, (f) PEI-8A.





**Figure S5.** Raman spectra of PEI-LMs composites.


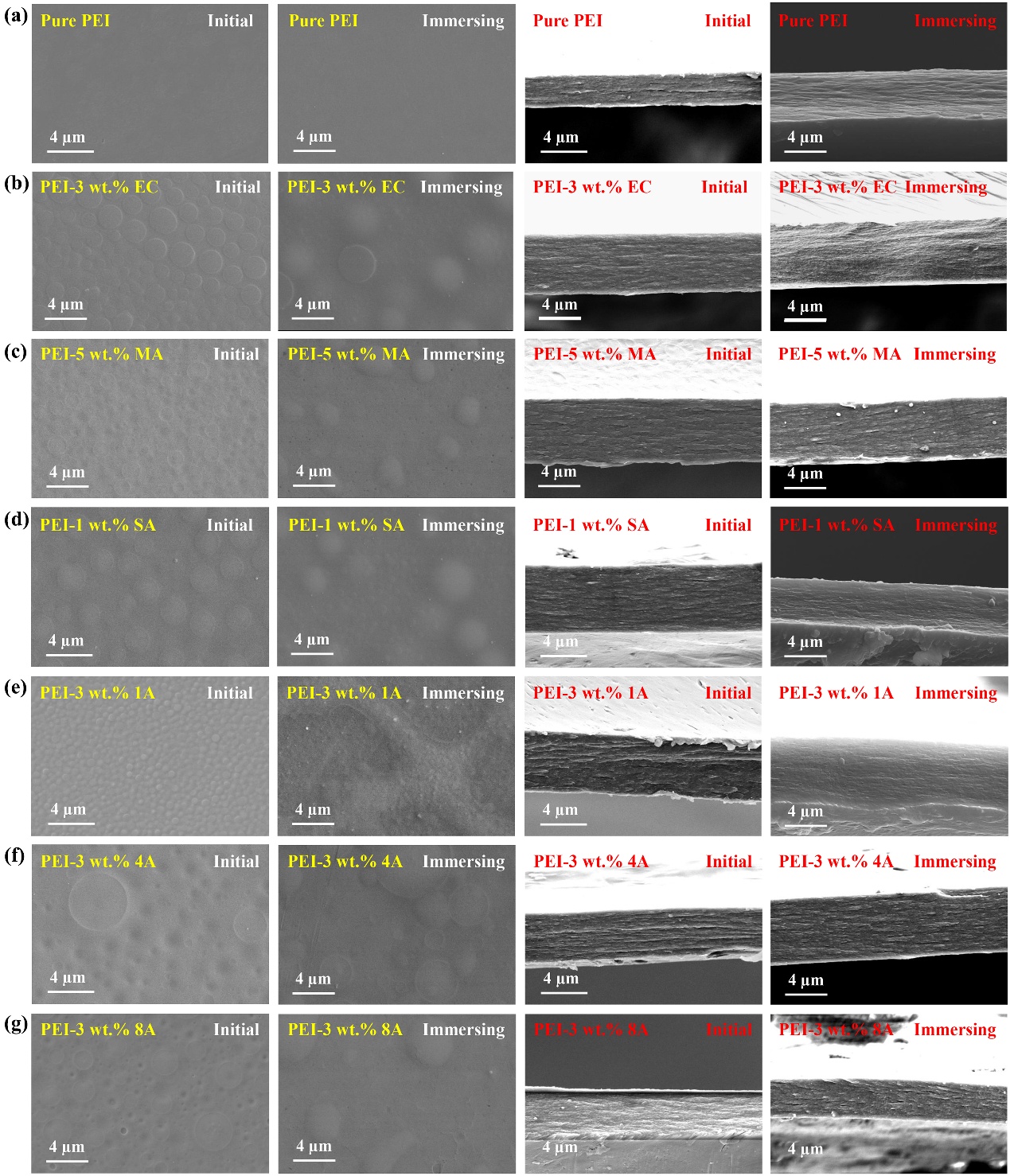


**Figure S6.** The surface and cross-section morphology of samples: (a) Pure PEI, (b) PEI-EC, (c) PEI-MA, (d) PEI-SA, (e) PEI-1A, (f) PEI-4A, (g) PEI-8A.


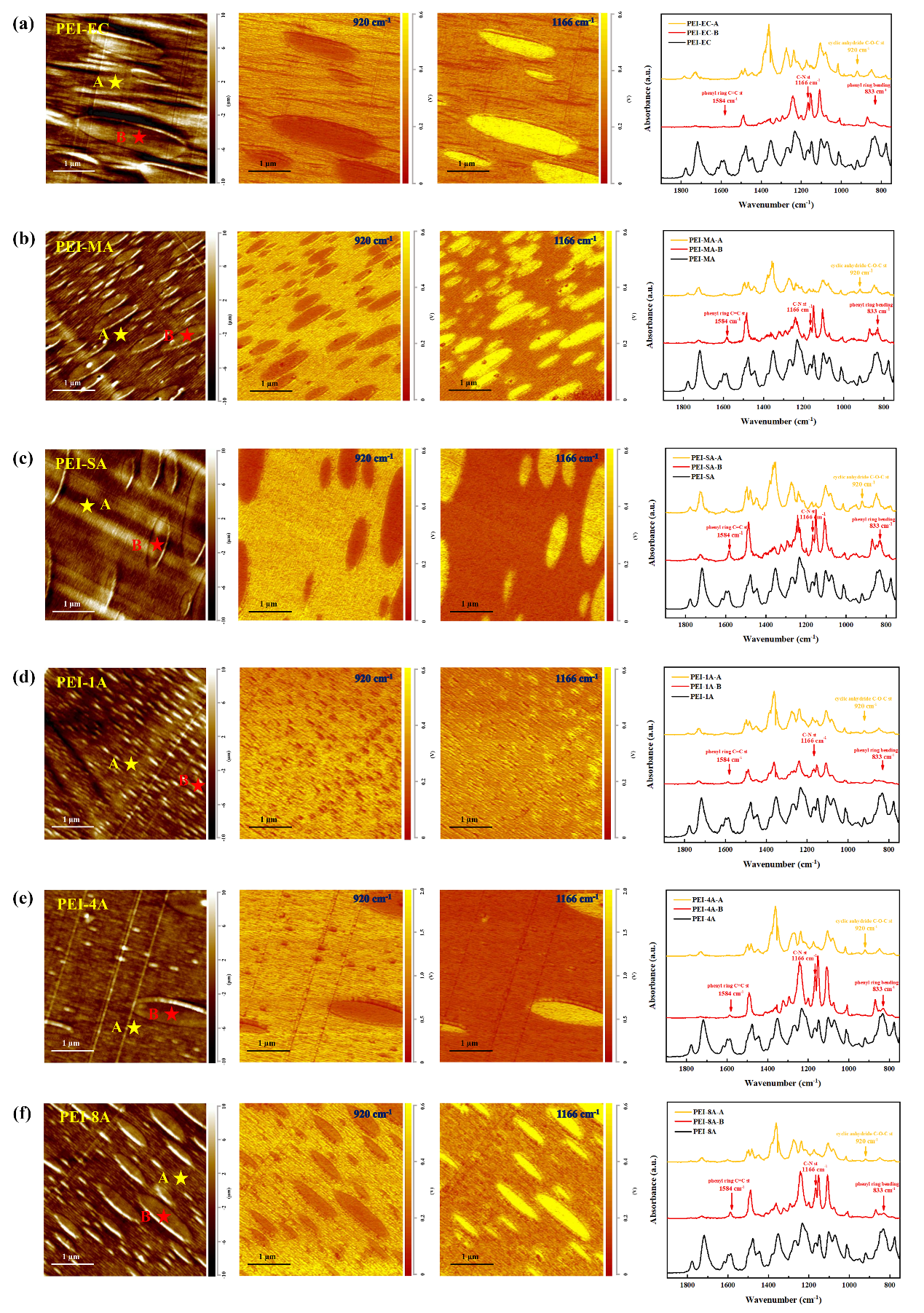


**Figure S7.** The AFM-IR chemical map with irradiation and corresponding height image of composites: (a) PEI-EC, (b) PEI-MA, (c) PEI-SA, (d) PEI-1A, (e) PEI-4A, (f) PEI-8A.

**Table S1.** The interchain spacing (nm) of samples calculated determined by XRD data.

|  | PEI-EC | PEI-MA | PEI-SA | PEI-1A | PEI-4A | PEI-8A |
| --- | --- | --- | --- | --- | --- | --- |
| 0 wt.% | 0.502 | 0.502 | 0.502 | 0.502 | 0.502 | 0.502 |
| 0.5 wt.% | - | - | 0.522 | - | - | - |
| 1.0 wt.% | 0.511 | 0.513 | 0.543 | 0.525 | 0.539 | 0.522 |
| 3.0 wt.% | 0.516 | 0.523 | 0.538 | 0.531 | 0.540 | 0.532 |
| 5.0 wt.% | 0.510 | 0.525 | 0.534 | 0.524 | 0.531 | 0.523 |
| 8.0 wt.% | 0.508 | 0.521 | 0.526 | 0.520 | 0.527 | 0.522 |
| 10.0 wt.% | 0.503 | 0.504 | 0.523 | 0.510 | 0.523 | 0.521 |

**Table S2.** The melting point *T_m_* of LMs.

| LMs | *T_m_* |
| --- | --- |
| EC | 35-38 ℃ |
| MA | 51-56 ℃ |
| SA | 118 ℃ |
| 1A | 33-35 °C |
| 4A | 44-48 °C |
| 8A | 63-67°C |


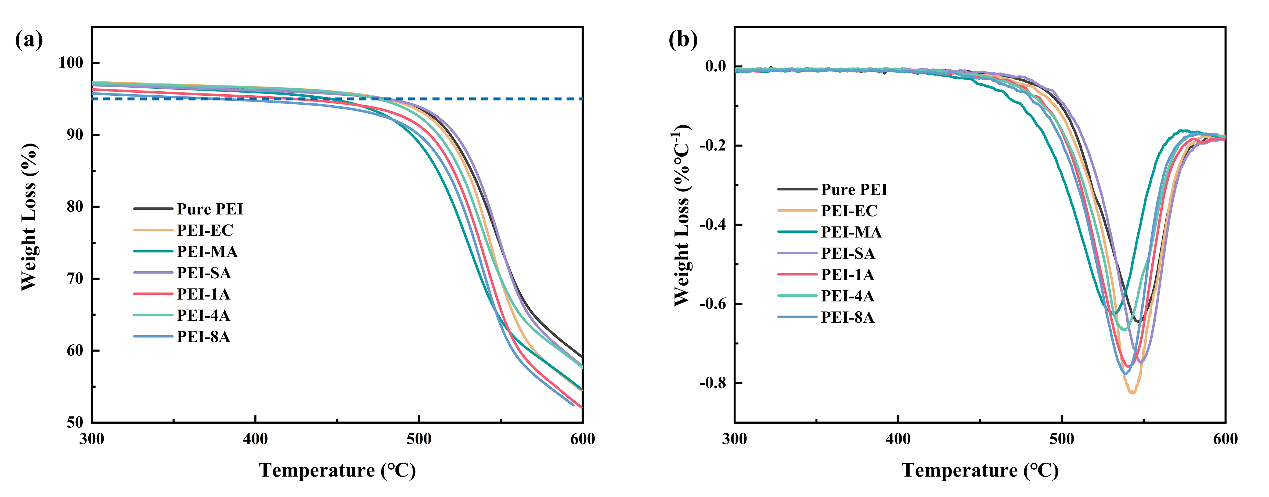


**Figure S8.** TGA and DTG curves of PEI-LMs composites.


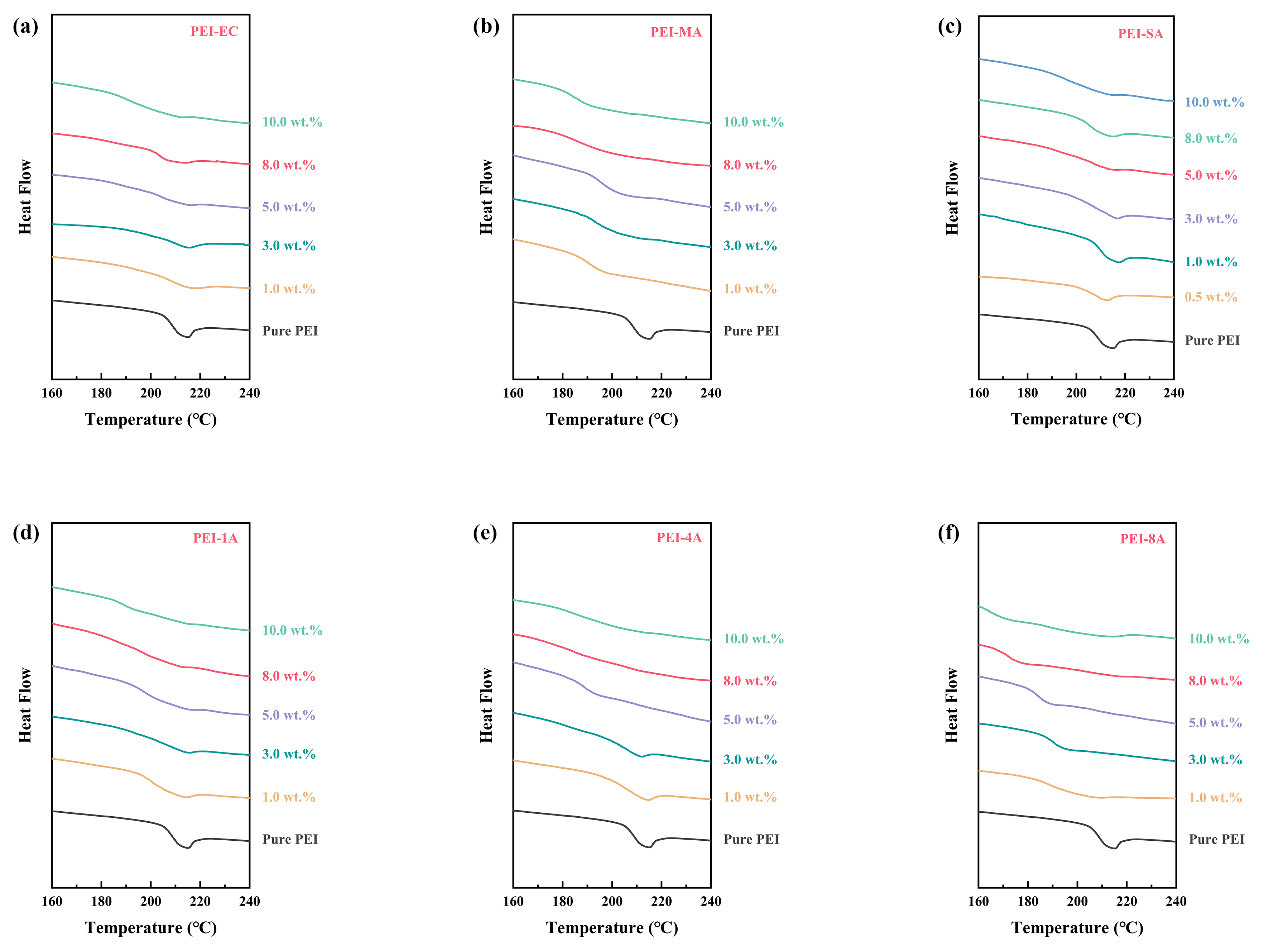


**Figure S9.** DSC data of composites: (a) PEI-EC, (b) PEI-MA, (c) PEI-SA, (d) PEI-1A, (e) PEI-4A, (f) PEI-8A.

**Table S3.** The glass transition temperature *T_g_* of PEI-LMs composites with different contents.

|  | PEI-EC | PEI-MA | PEI-SA | PEI-1A | PEI-4A | PEI-8A |
| --- | --- | --- | --- | --- | --- | --- |
| 0 wt.% | 216.92 | 216.92 | 216.92 | 216.92 | 216.92 | 216.92 |
| 0.5 wt.% | - | - | 208.79 | - | - | - |
| 1.0 wt.% | 206.76 | 190.62 | 211.06 | 200.20 | 204.39 | 188.82 |
| 3.0 wt.% | 206.94 | 192.15 | 206.94 | 204.82 | 205.64 | 190.38 |
| 5.0 wt.% | 204.40 | 194.79 | 206.12 | 197.03 | 188.49 | 183.97 |
| 8.0 wt.% | 202.71 | 186.69 | 204.58 | 196.76 | 185.15 | 171.89 |
| 10.0 wt.% | 190.98 | 185.53 | 195.12 | 188.80 | 183.8 | 164.77 |





**Figure S10.** TMA curves of PEI-LMs composites.


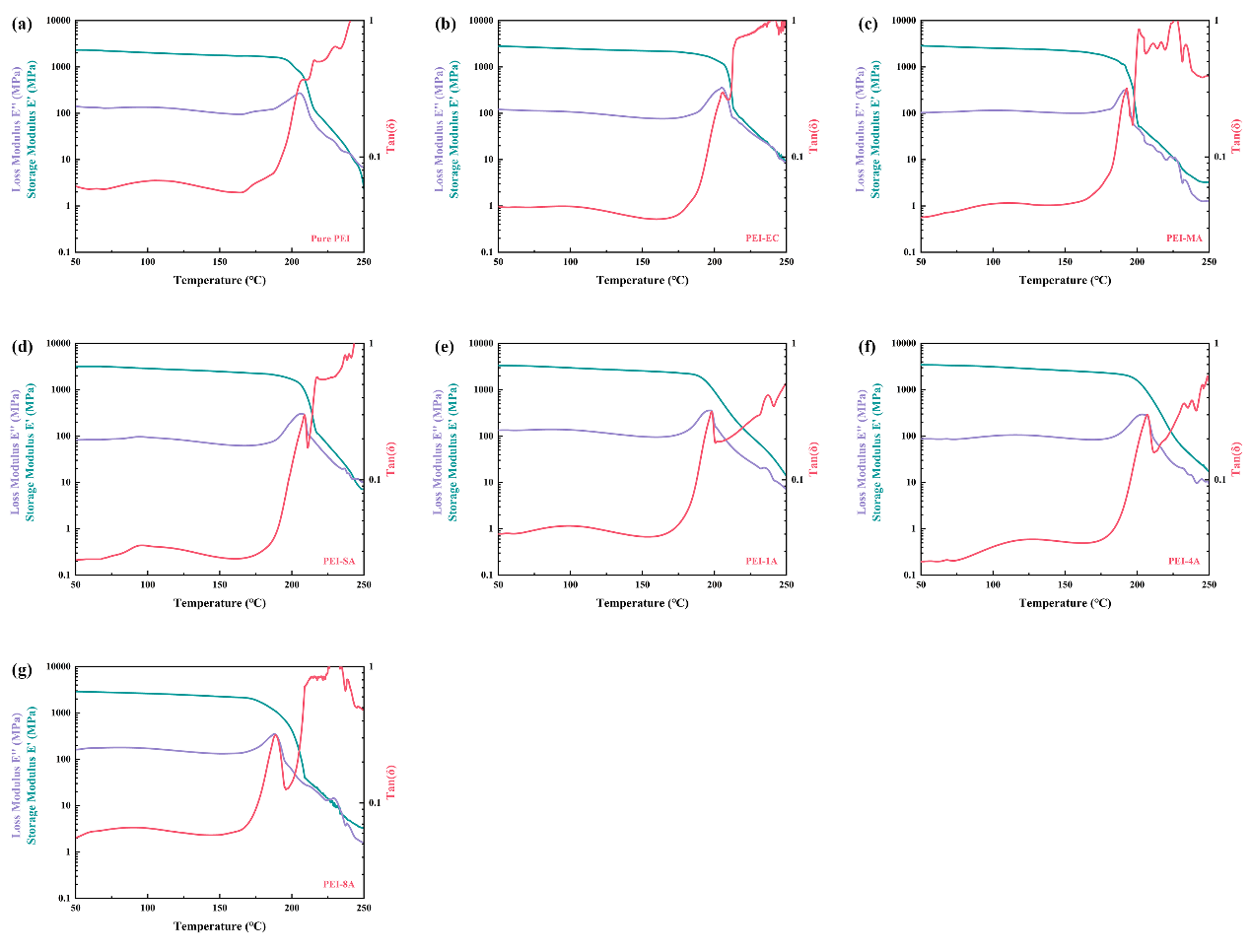


**Figure S11.** DMA curves of samples: (a) Pure PEI, (b) PEI-EC, (c) PEI-MA, (d) PEI-SA, (e) PEI-1A, (f) PEI-4A, (g) PEI-8A.





**Figure S12.** Tensile strain curves of PEI-LMs composites.


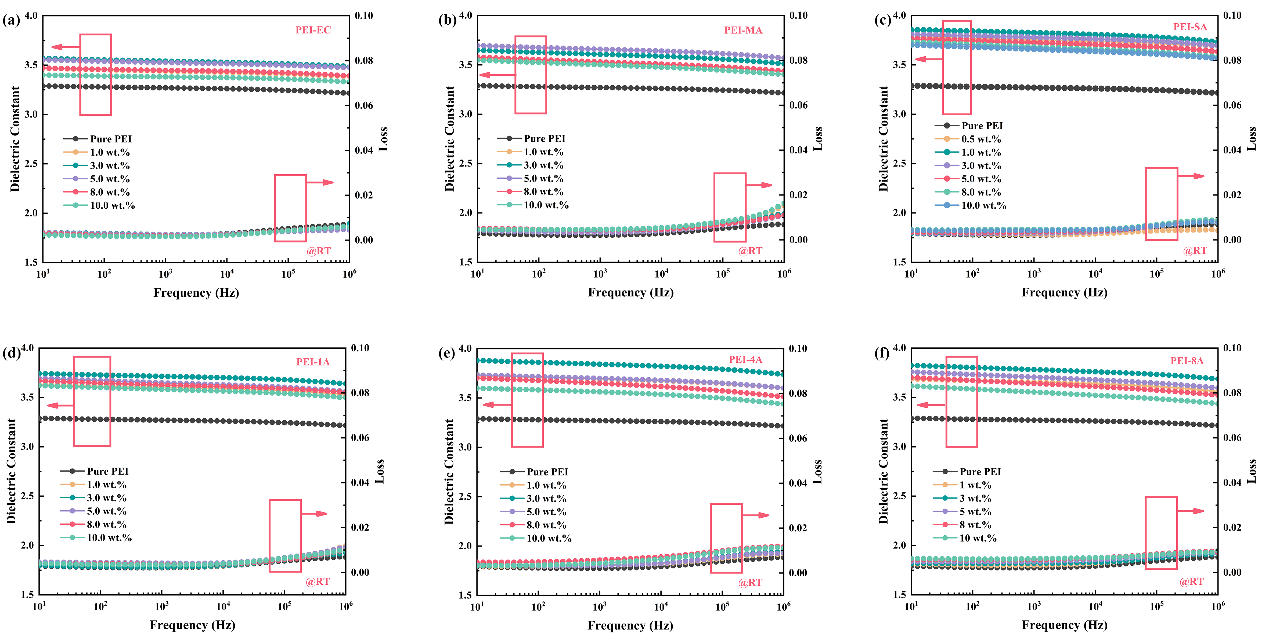


**Figure S13.** Dielectric frequency spectra of PEI-LMs composites: (a) PEI-EC, (b) PEI-MA, (c) PEI-SA, (d) PEI-1A, (e) PEI-4A, (f) PEI-8A.


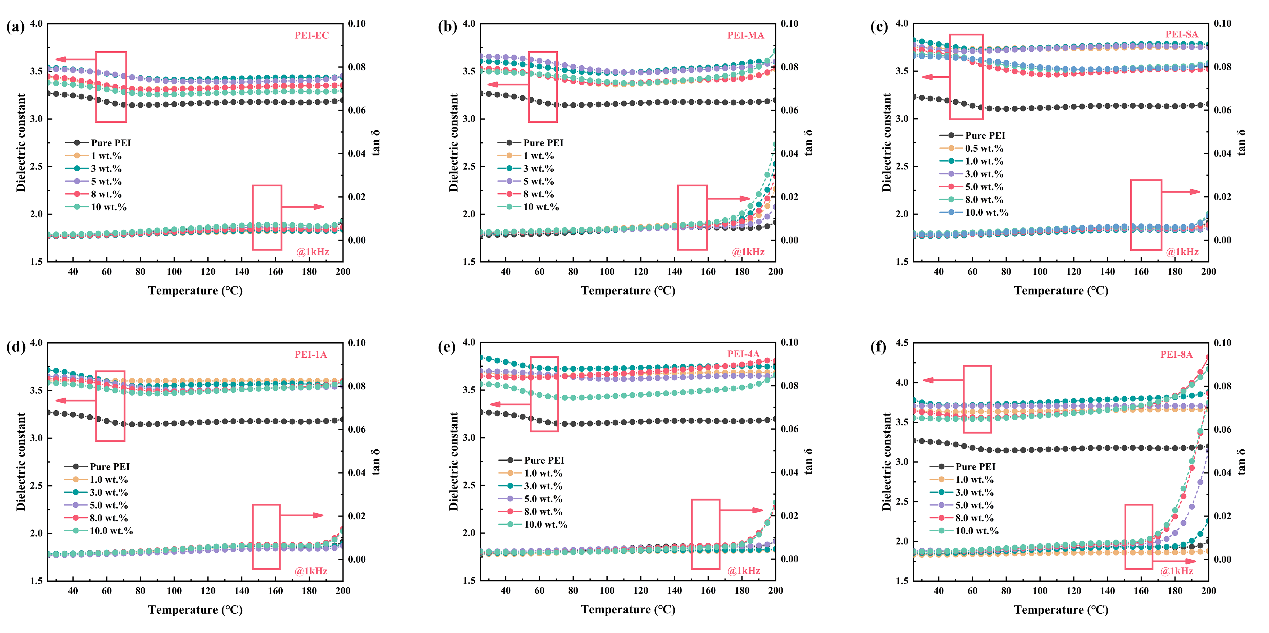


**Figure S14.** Dielectric temperature spectra of PEI-LMs composites: (a) PEI-EC, (b) PEI-MA, (c) PEI-SA, (d) PEI-1A, (e) PEI-4A, (f) PEI-8A.


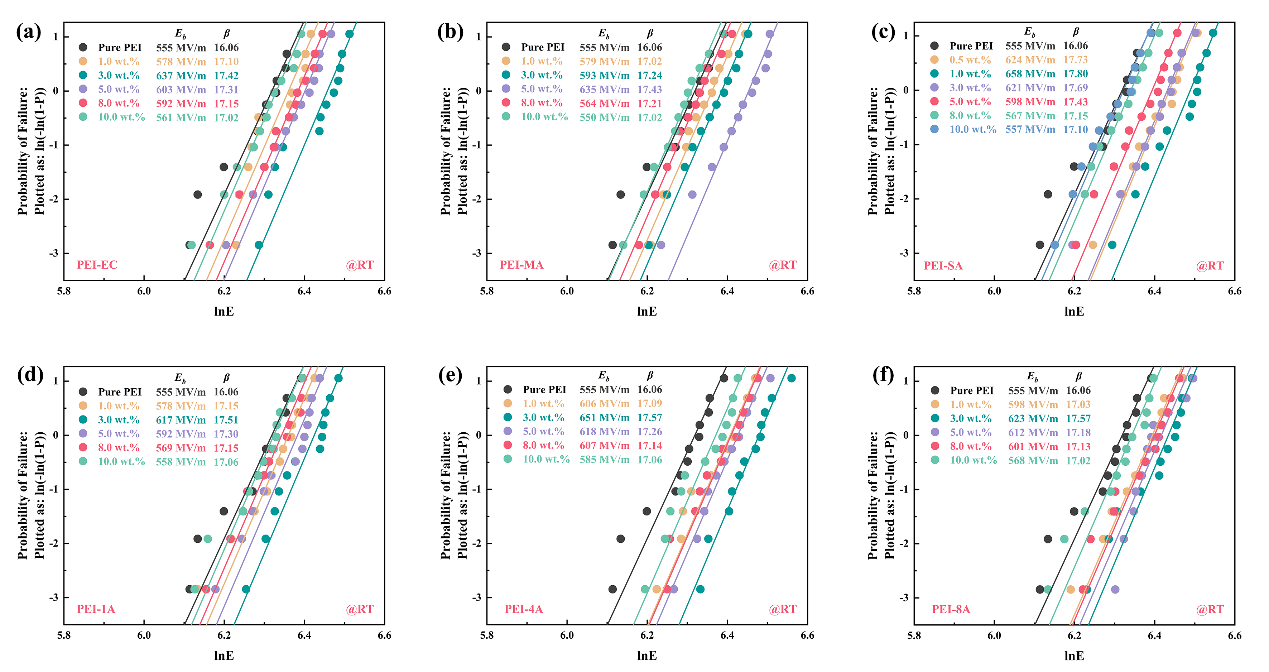


**Figure S15.** Weibull distribution of breakdown strength of PEI-LMs composites at room temperature: (a) PEI-EC, (b) PEI-MA, (c) PEI-SA, (d) PEI-1A, (e) PEI-4A, (f) PEI-8A.


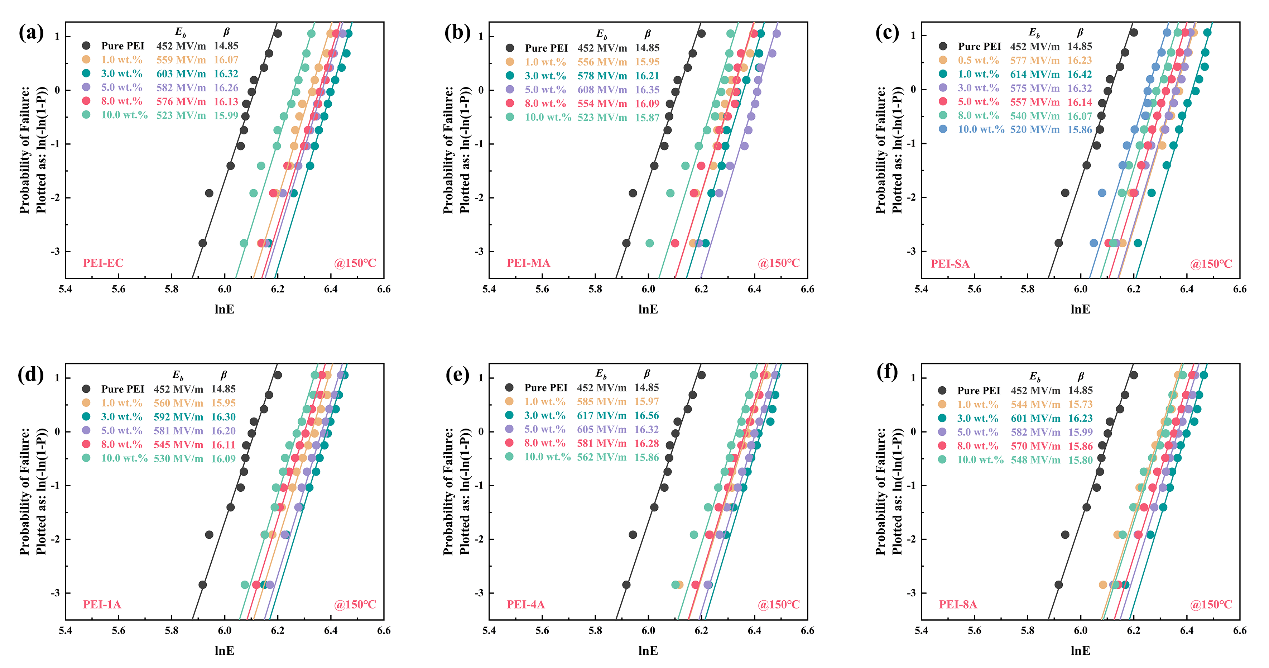


**Figure S16.** Weibull distribution of breakdown strength of PEI-LMs composites at 150 ℃: (a) PEI-EC, (b) PEI-MA, (c) PEI-SA, (d) PEI-1A, (e) PEI-4A, (f) PEI-8A.


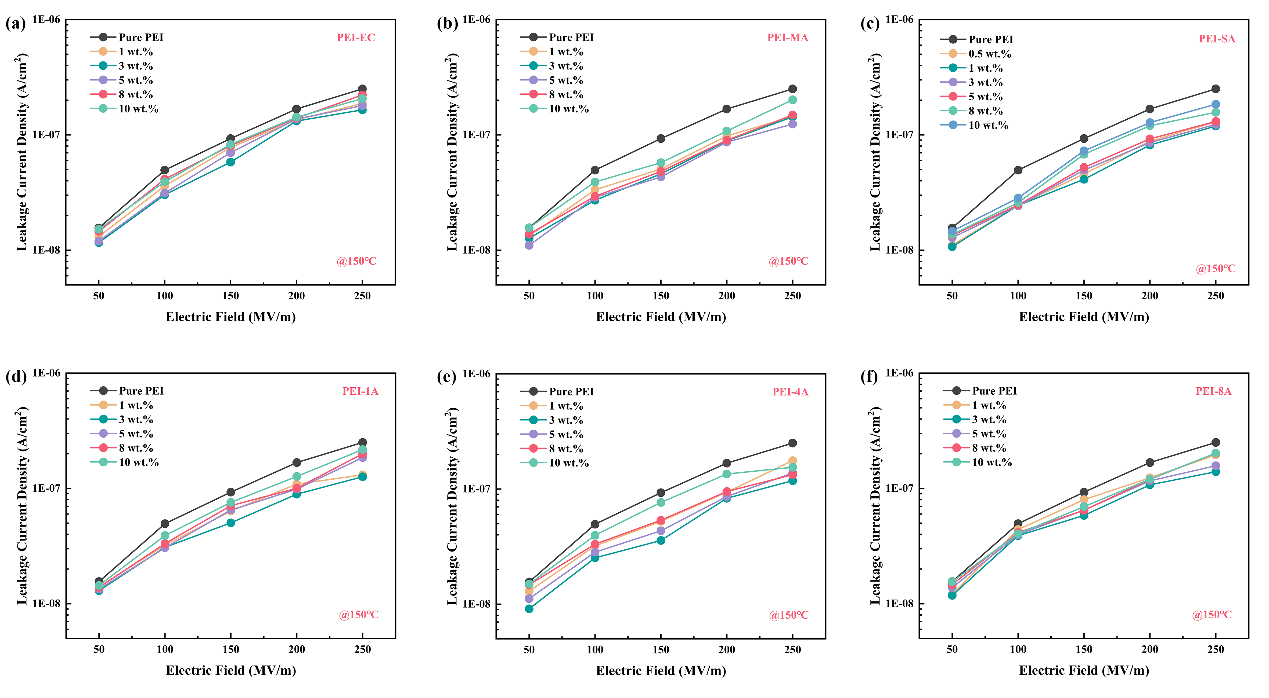


**Figure S17.** Leakage current density versus electric field of PEI-LMs composites at 150 ℃: (a) PEI-EC, (b) PEI-MA, (c) PEI-SA, (d) PEI-1A, (e) PEI-4A, (f) PEI-8A.


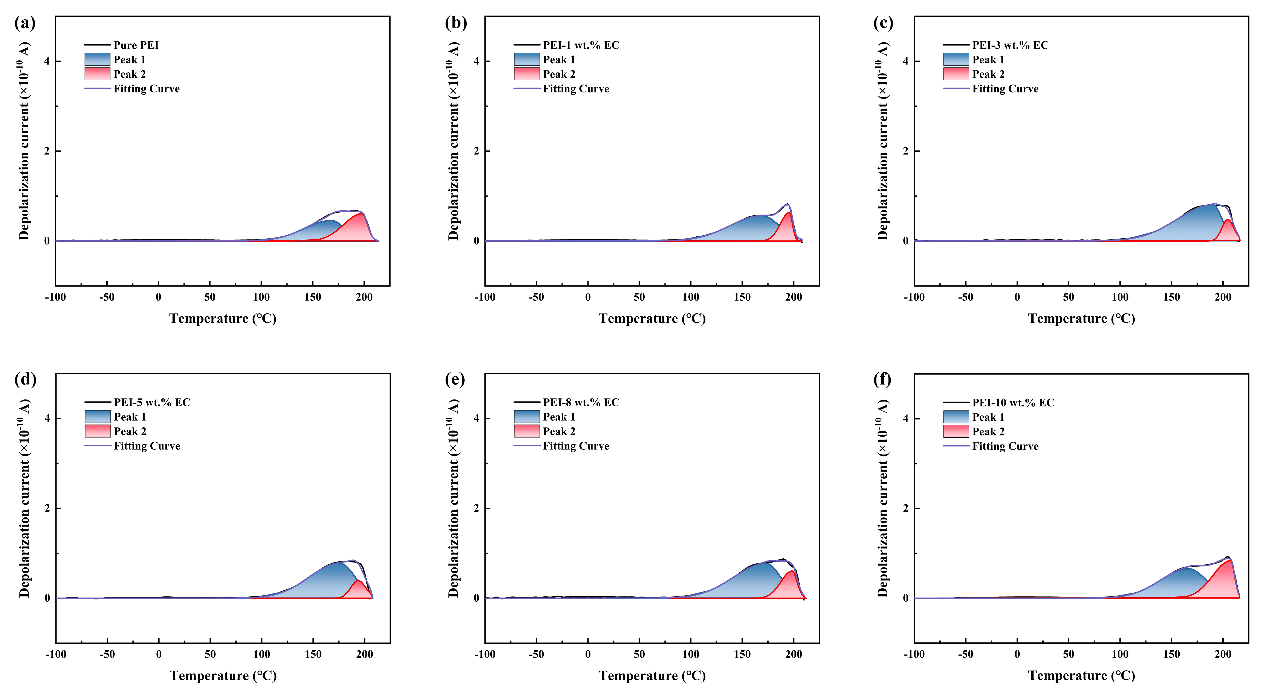


**Figure S18.** TSDC curves of PEI-EC. (a) Pure PEI, (b) 1.0 wt.%, (c) 3.0 wt.%, (d) 5.0 wt.%, (e) 8.0 wt.%, (f) 10.0 wt.%.


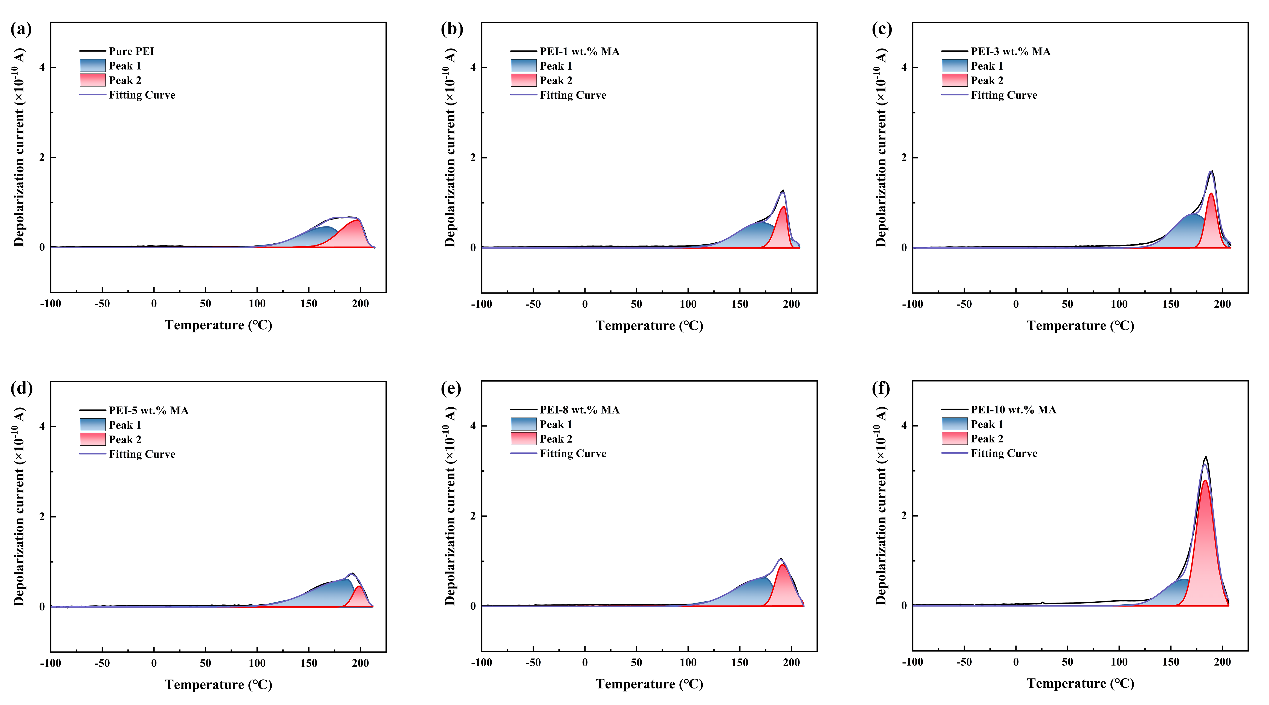


**Figure S19.** TSDC curves of PEI-MA. (a) Pure PEI, (b) 1.0 wt.%, (c) 3.0 wt.%, (d) 5.0 wt.%, (e) 8.0 wt.%, (f) 10.0 wt.%.


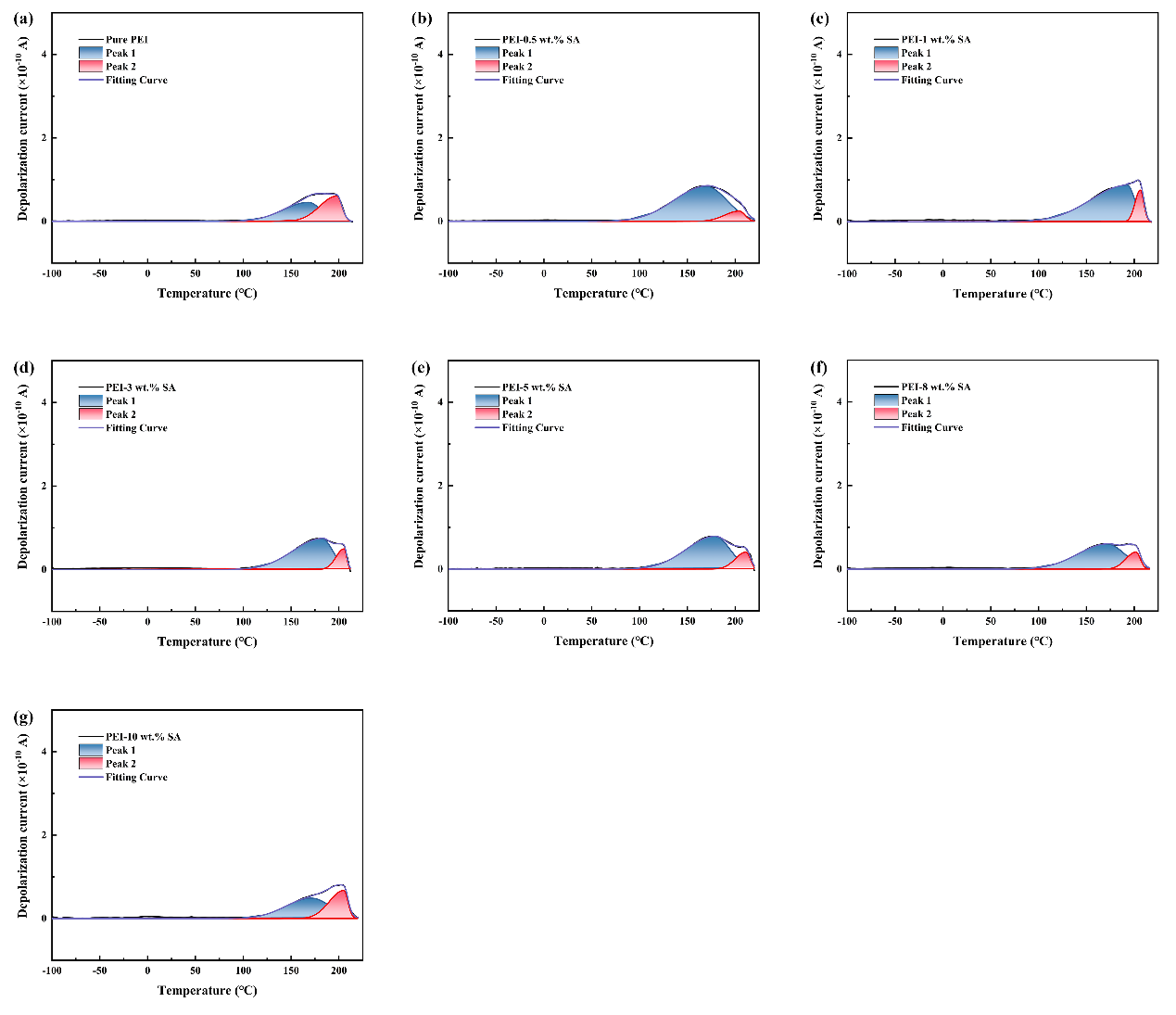


**Figure S20.** TSDC curves of PEI-SA. (a) Pure PEI, (b) 0.5 wt.%, (c) 1.0 wt.%, (d) 3.0 wt.%, (e) 5.0 wt.%, (f) 8.0 wt.%, (g) 10.0 wt.%.


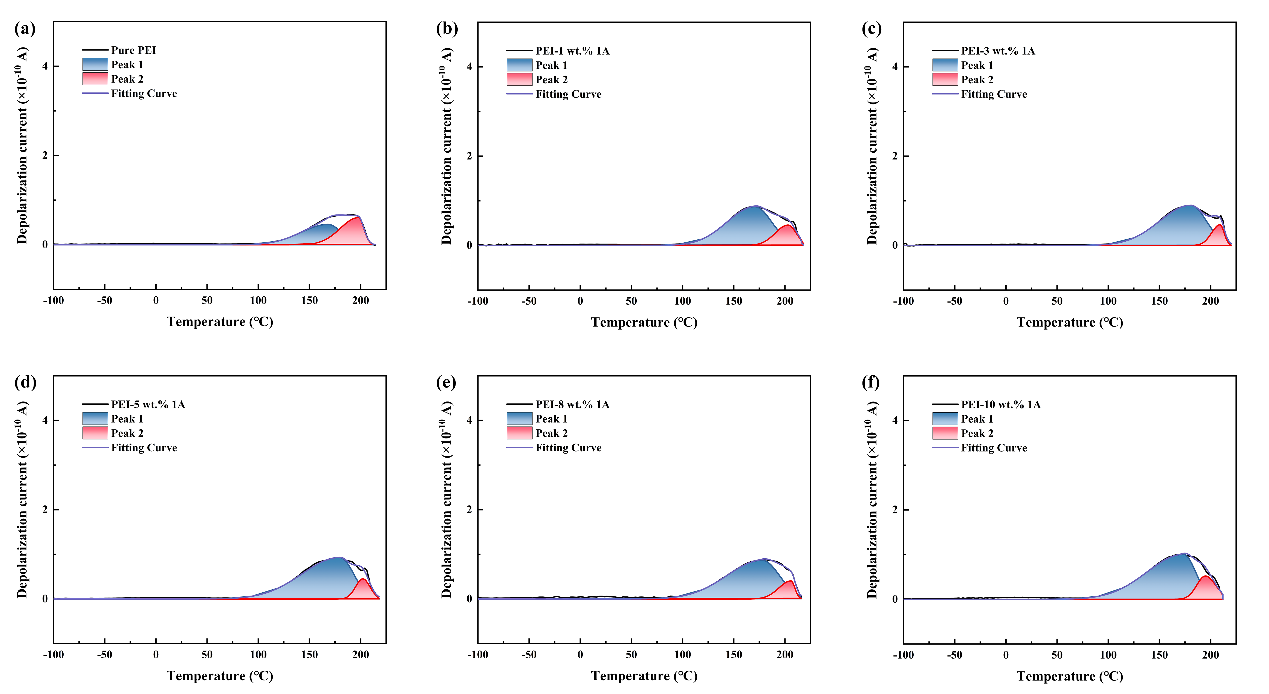


**Figure S21.** TSDC curves of PEI-1A. (a) Pure PEI, (b) 1.0 wt.%, (c) 3.0 wt.%, (d) 5.0 wt.%, (e) 8.0 wt.%, (f) 10.0 wt.%.


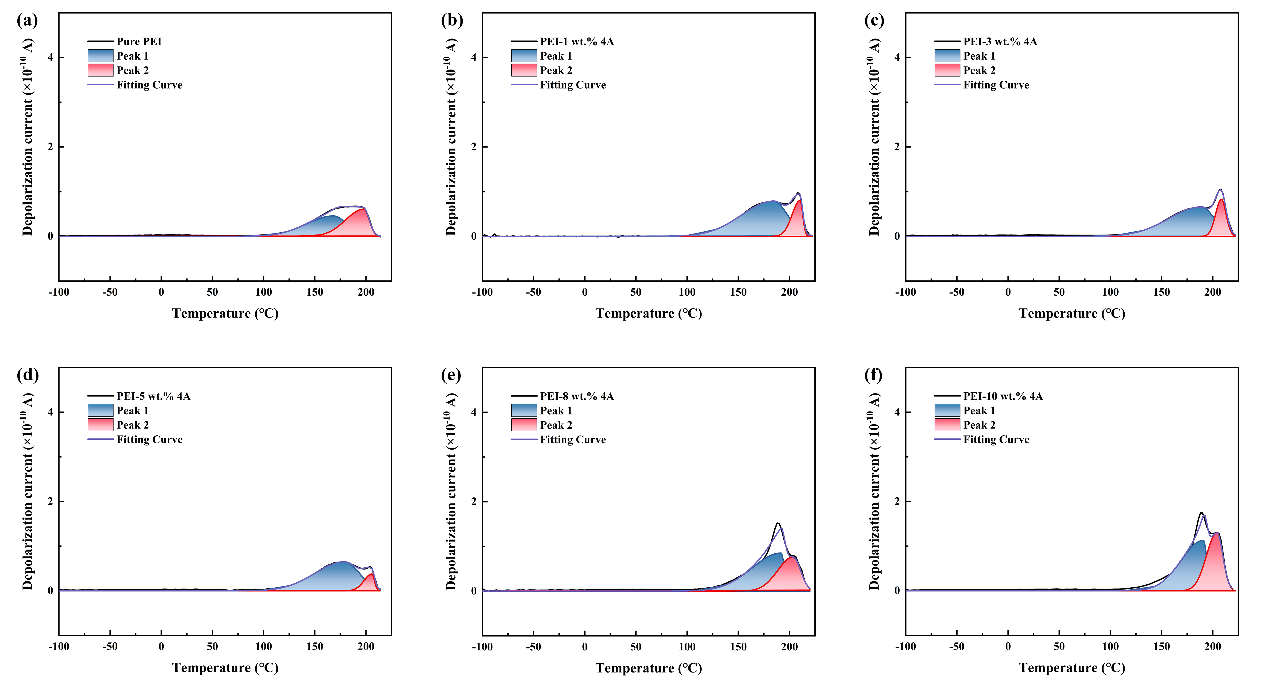


**Figure S22.** TSDC curves of PEI-4A. (a) Pure PEI, (b) 1.0 wt.%, (c) 3.0 wt.%, (d) 5.0 wt.%, (e) 8.0 wt.%, (f) 10.0 wt.%.


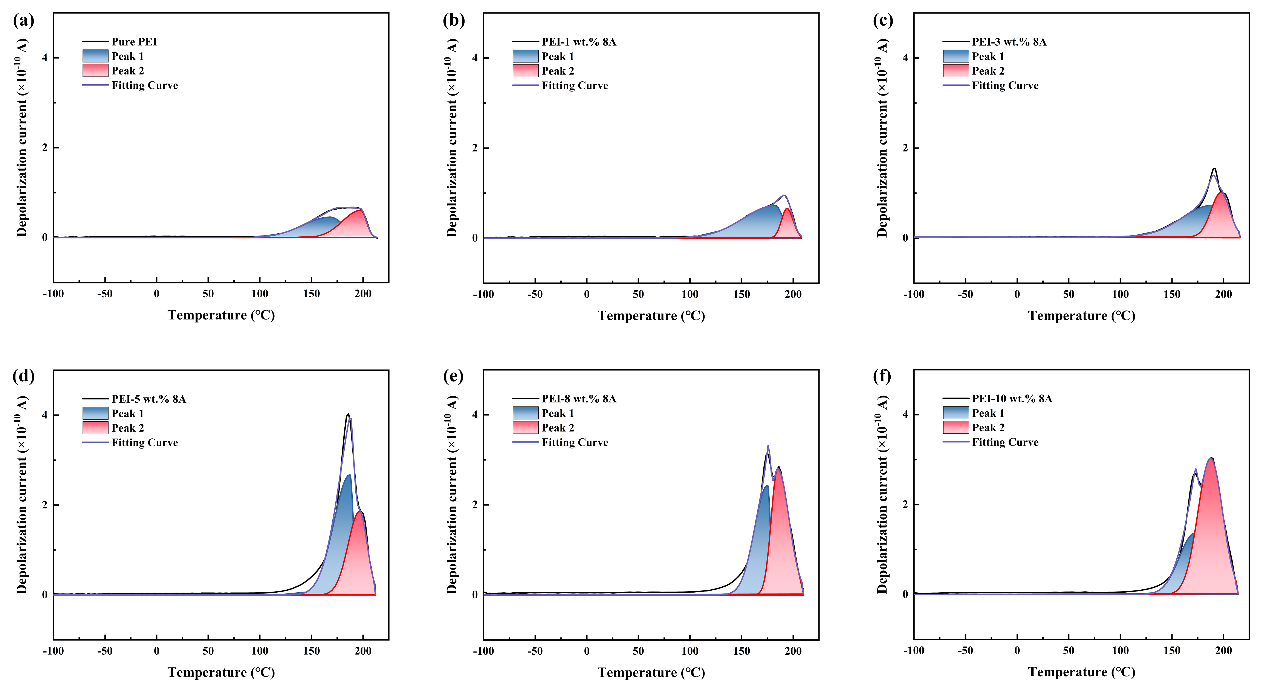


**Figure S23.** TSDC curves of PEI-8A. (a) Pure PEI, (b) 1.0 wt.%, (c) 3.0 wt.%, (d) 5.0 wt.%, (e) 8.0 wt.%, (f) 10.0 wt.%.

**Table S4.** Trap depth (eV) determined by TSDC peaks of PEI-LMs composites.

|  | Content | PEI-EC | PEI-MA | PEI-SA | PEI-1A | PEI-4A | PEI-8A |
| --- | --- | --- | --- | --- | --- | --- | --- |
| Peak 1 | 0 wt.% | 0.810 | 0.810 | 0.810 | 0.810 | 0.810 | 0.810 |
|  | 0.5 wt.% | - | - | 0.692 | - | - | - |
|  | 1.0 wt.% | 0.709 | 0.789 | 0.853 | 0.782 | 0.805 | 0.932 |
|  | 3.0 wt.% | 0.840 | 0.911 | 0.860 | 0.777 | 0.827 | 1.114 |
|  | 5.0 wt.% | 0.781 | 0.921 | 0.774 | 0.705 | 0.812 | 1.957 |
|  | 8.0 wt.% | 0.753 | 0.911 | 0.701 | 0.684 | 1.177 | 1.910 |
|  | 10.0 wt.% | 0.742 | 0.825 | 0.733 | 0.683 | 1.488 | 1.888 |
| Peak 2 | 0 wt.% | 1.605 | 1.605 | 1.605 | 1.605 | 1.605 | 1.605 |
|  | 0.5 wt.% | - | - | 1.946 | - | - | - |
|  | 1.0 wt.% | 3.232 | 3.367 | 4.395 | 1.913 | 3.903 | 3.365 |
|  | 3.0 wt.% | 3.380 | 3.474 | 3.563 | 3.285 | 3.966 | 2.135 |
|  | 5.0 wt.% | 2.591 | 3.487 | 2.625 | 2.797 | 3.874 | 2.068 |
|  | 8.0 wt.% | 2.451 | 2.288 | 2.612 | 2.520 | 1.763 | 1.661 |
|  | 10.0 wt.% | 1.899 | 2.109 | 2.047 | 2.052 | 2.463 | 1.461 |

**Table S5.** Trap density (10^18^ m^3^) determined by TSDC peaks of PEI-LMs composites.

|  | Content | PEI-EC | PEI-MA | PEI-SA | PEI-1A | PEI-4A | PEI-8A |
| --- | --- | --- | --- | --- | --- | --- | --- |
| Peak 1 | 0 wt.% | 2.60 | 2.60 | 2.60 | 2.60 | 2.60 | 2.60 |
|  | 0.5 wt.% | - | - | 4.56 | - | - | - |
|  | 1.0 wt.% | 3.36 | 1.25 | 4.29 | 5.42 | 5.02 | 4.01 |
|  | 3.0 wt.% | 5.58 | 3.58 | 4.13 | 6.32 | 5.25 | 4.19 |
|  | 5.0 wt.% | 5.72 | 3.75 | 4.20 | 6.45 | 4.69 | 6.62 |
|  | 8.0 wt.% | 4.86 | 3.03 | 4.08 | 6.27 | 4.44 | 3.73 |
|  | 10.0 wt.% | 3.75 | 2.48 | 2.41 | 5.96 | 4.06 | 3.52 |
| Peak 2 | 0 wt.% | 2.00 | 2.00 | 2.00 | 2.00 | 2.00 | 2.00 |
|  | 0.5 wt.% | - | - | 0.73 | - | - | - |
|  | 1.0 wt.% | 0.93 | 2.99 | 0.74 | 1.31 | 1.17 | 1.05 |
|  | 3.0 wt.% | 0.87 | 1.69 | 0.74 | 0.87 | 1.50 | 3.17 |
|  | 5.0 wt.% | 0.90 | 0.80 | 0.70 | 0.90 | 0.64 | 4.48 |
|  | 8.0 wt.% | 1.30 | 1.88 | 0.86 | 0.90 | 2.78 | 8.79 |
|  | 10.0 wt.% | 2.22 | 5.15 | 1.39 | 1.38 | 2.97 | 11.1 |


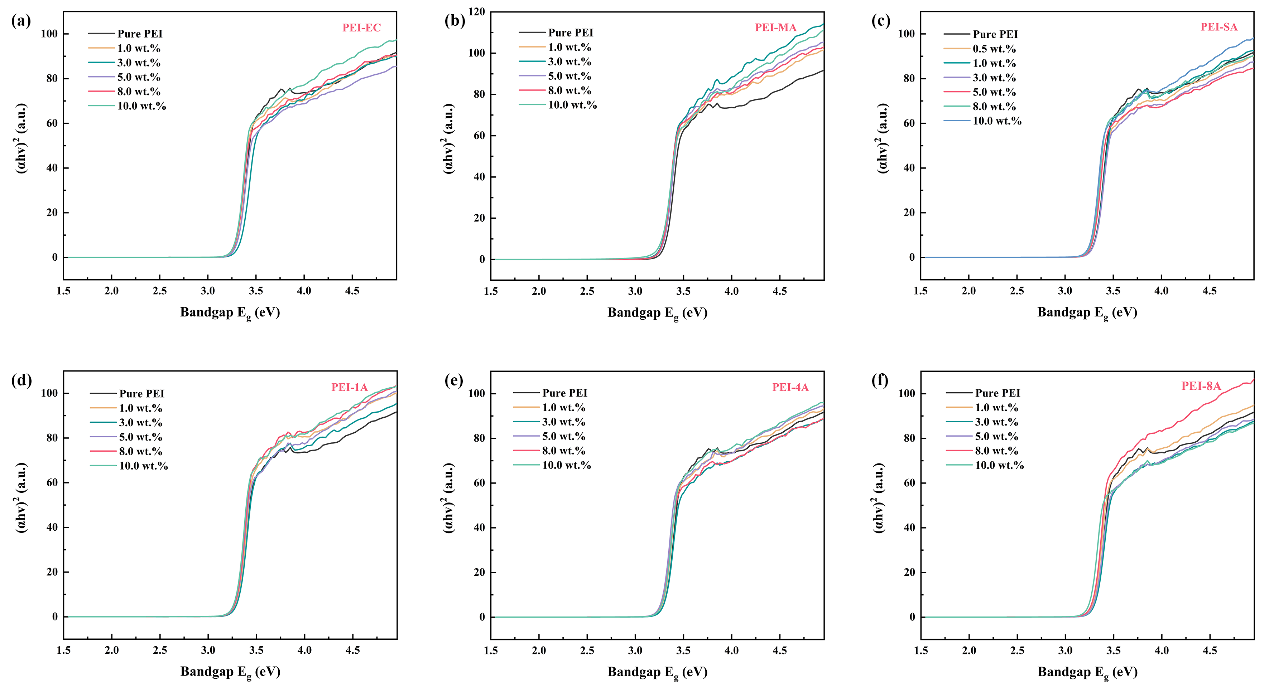


**Figure S24.** UV-visible spectra of PEI-LMs composites: (a) PEI-EC, (b) PEI-MA, (c) PEI-SA, (d) PEI-1A, (e) PEI-4A, (f) PEI-8A.

**Table S6.** The bandgap *E_g_* (eV) of PEI-LMs composites with different contents.

|  | PEI-EC | PEI-MA | PEI-SA | PEI-1A | PEI-4A | PEI-8A |
| --- | --- | --- | --- | --- | --- | --- |
| 0 wt.% | 3.318 | 3.318 | 3.318 | 3.318 | 3.318 | 3.318 |
| 0.5 wt.% | - | - | 3.314 | - | - | - |
| 1.0 wt.% | 3.301 | 3.288 | 3.318 | 3.315 | 3.301 | 3.302 |
| 3.0 wt.% | 3.345 | 3.292 | 3.316 | 3.323 | 3.313 | 3.319 |
| 5.0 wt.% | 3.313 | 3.303 | 3.295 | 3.303 | 3.282 | 3.308 |
| 8.0 wt.% | 3.295 | 3.294 | 3.282 | 3.303 | 3.293 | 3.299 |
| 10.0 wt.% | 3.295 | 3.280 | 3.274 | 3.294 | 3.294 | 3.260 |


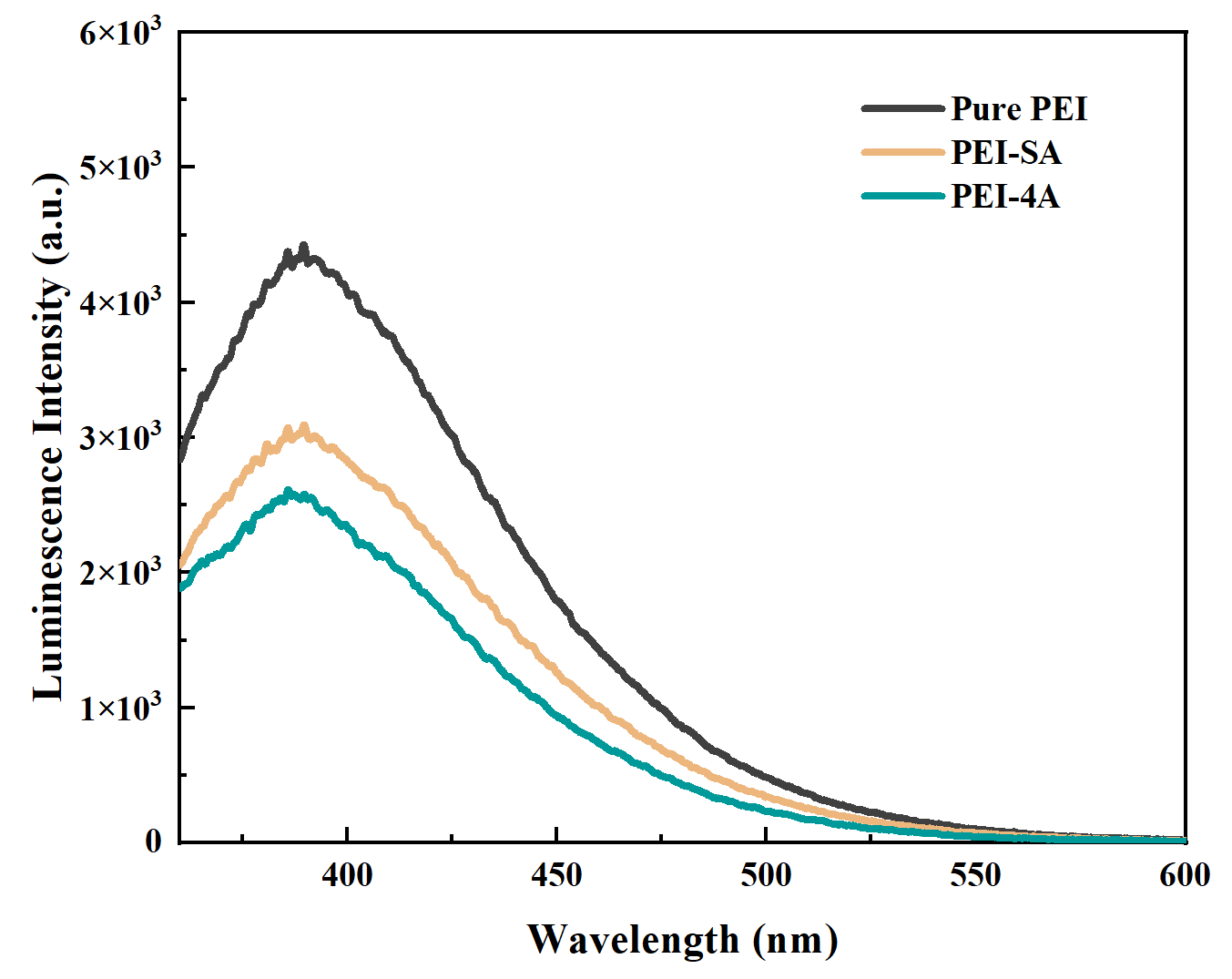


**Figure S25**. The emission spectra excited at 325 nm of different composites measured by photoluminescence spectroscopy.

**Table S7.** The EA and IE calculated for different LMs.

|  | EA (eV) | -IE (eV) |
| --- | --- | --- |
| EC | -3.62 | -10.88 |
| MA | 0.76 | -10.70 |
| SA | -1.52 | -10.28 |
| 1A | -1.43 | -10.13 |
| 4A | -1.37 | -9.78 |
| 8A | -1.35 | -9.51 |


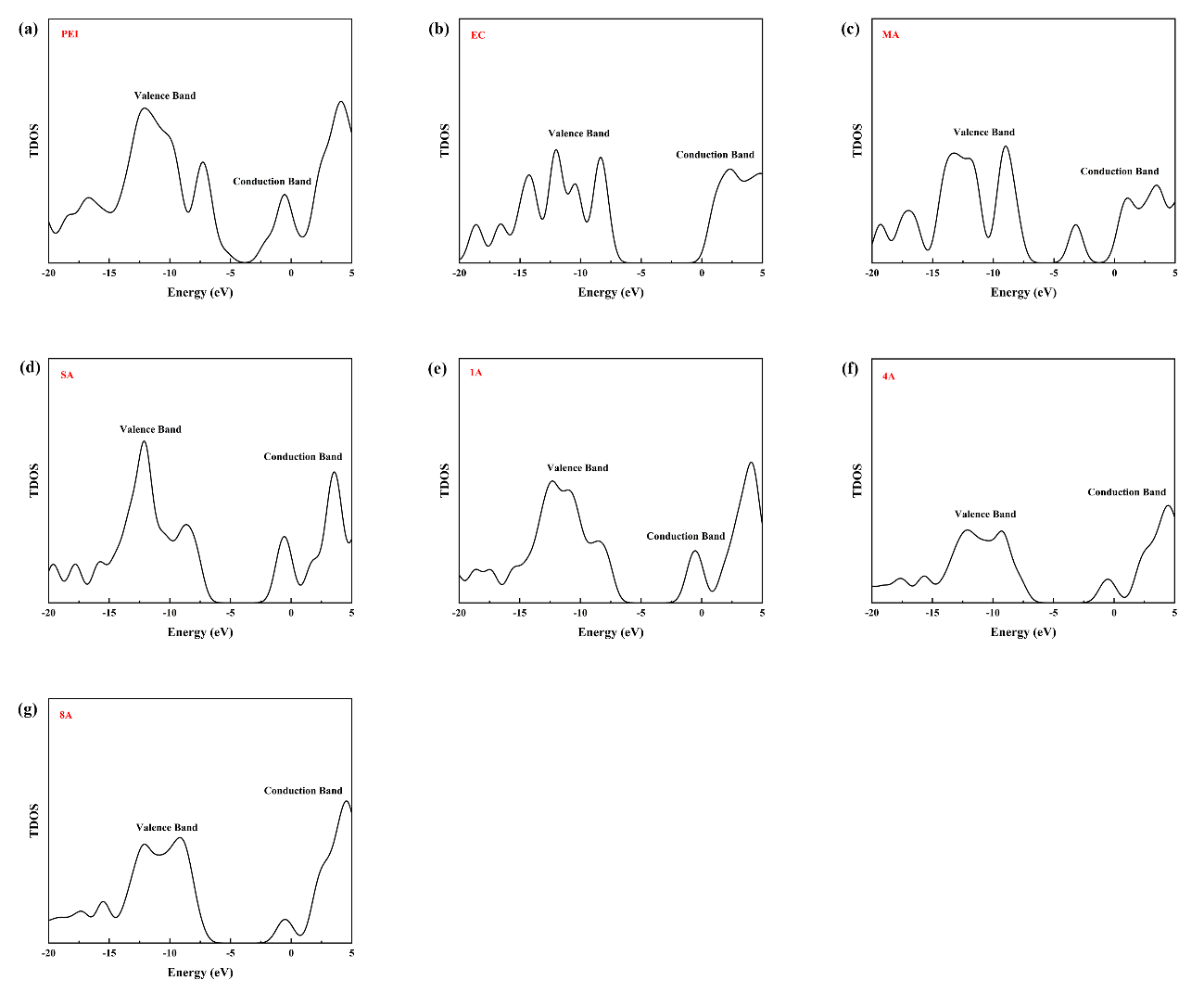


**Figure S26**. Calculated electronic density of states of (a) PEI and (b) EC, (c) MA, (d) SA, (e) 1A, (f) 4A, (g) 8A.


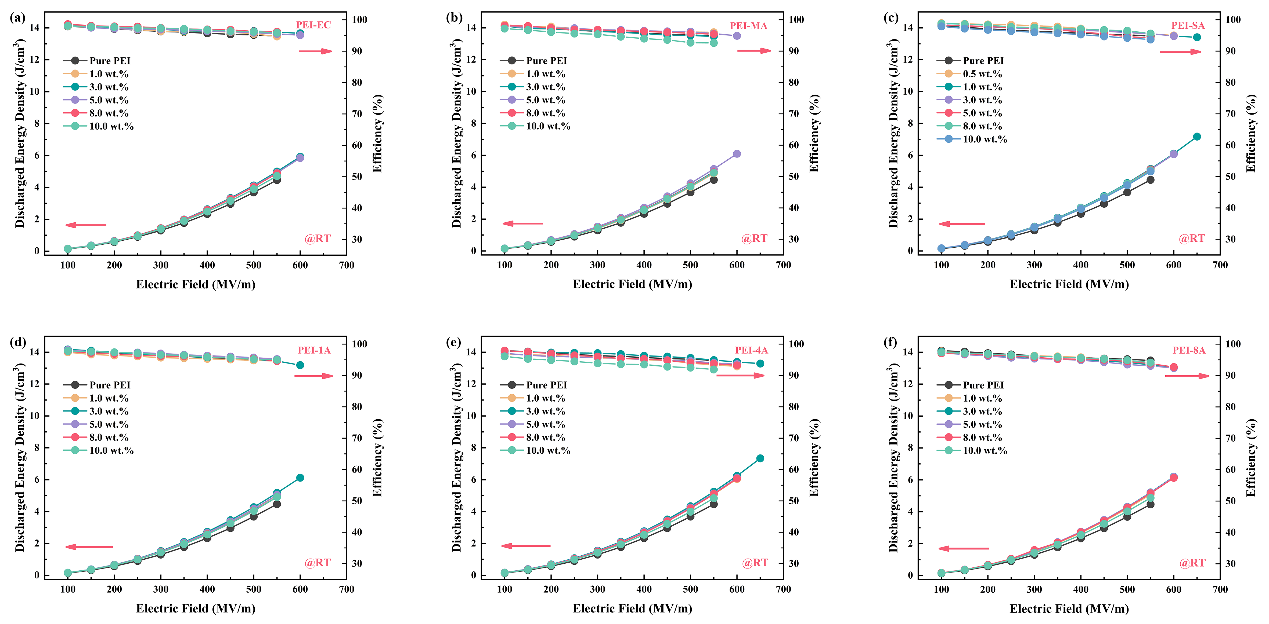


**Figure S27.** Discharged energy density and efficiency versus electric field of PEI-LMs composites at room temperature: (a) PEI-EC, (b) PEI-MA, (c) PEI-SA, (d) PEI-1A, (e) PEI-4A, (f) PEI-8A.


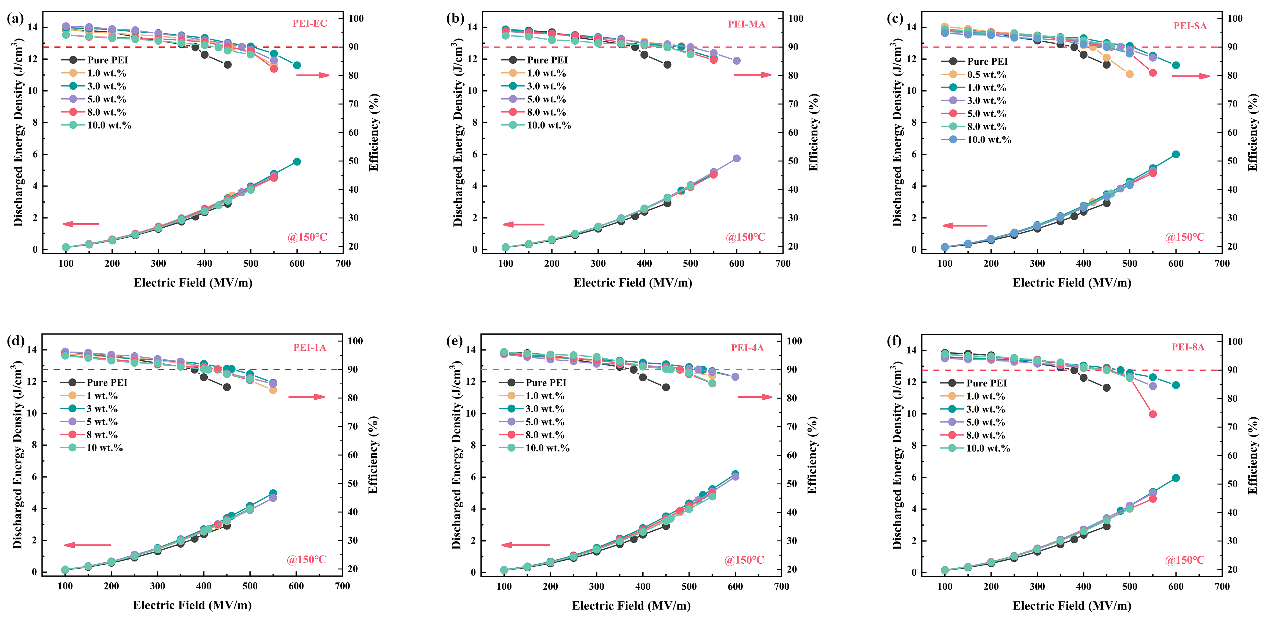


**Figure S28.** Discharged energy density and efficiency versus electric field of PEI-LMs composites at 150 ℃: (a) PEI-EC, (b) PEI-MA, (c) PEI-SA, (d) PEI-1A, (e) PEI-4A, (f) PEI-8A.


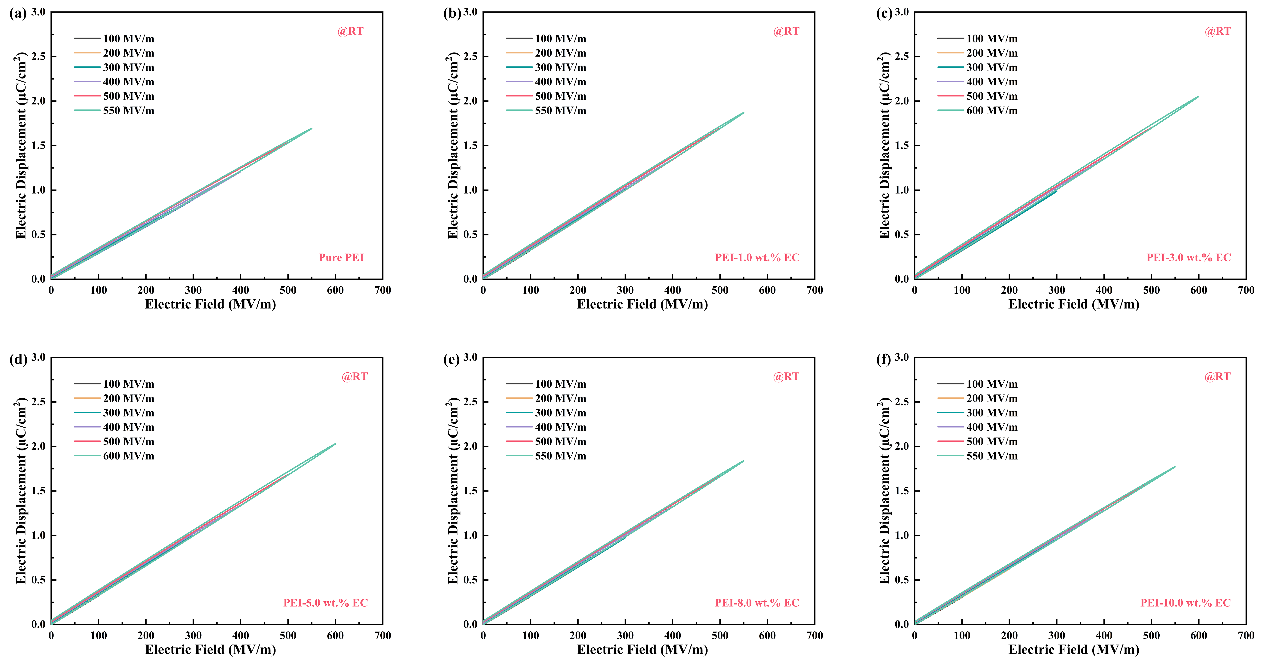


**Figure S29.** *D-E* loops of PEI-EC at room temperature: (a) Pure PEI, (b) 1.0 wt.%, (c) 3.0 wt.%, (d) 5.0 wt.%, (e) 8.0 wt.%, (f) 10.0 wt.%.


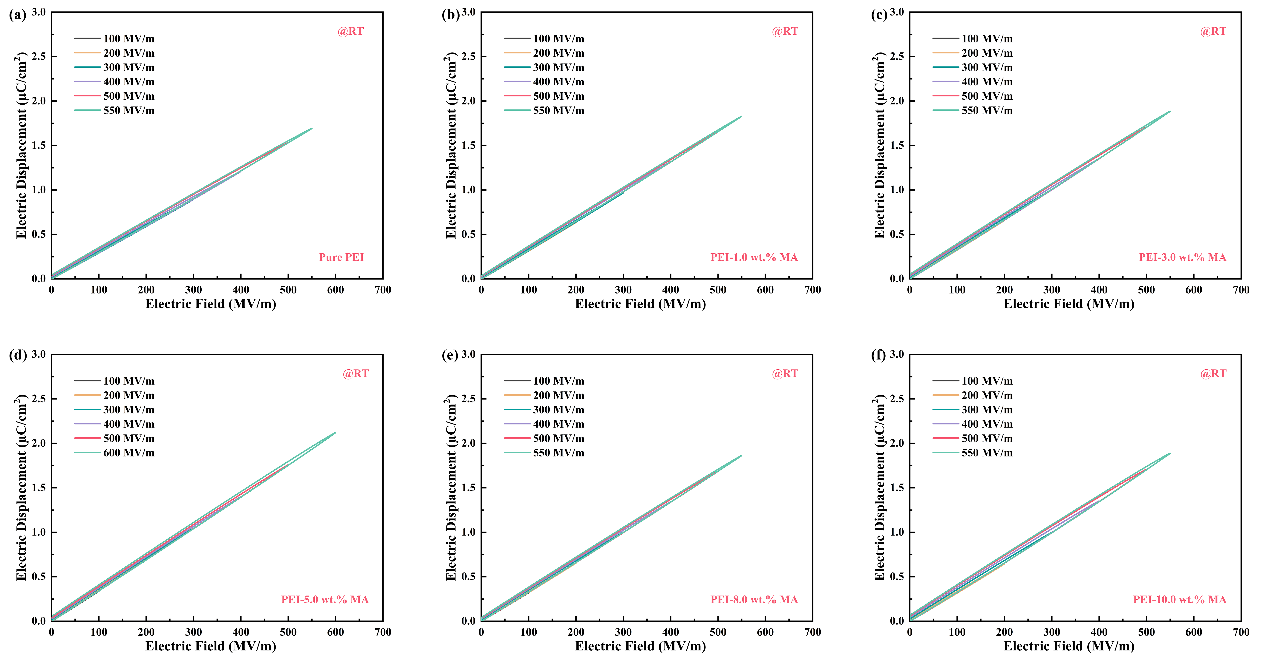


**Figure S30.** *D-E* loops of PEI-MA at room temperature: (a) Pure PEI, (b) 1.0 wt.%, (c) 3.0 wt.%, (d) 5.0 wt.%, (e) 8.0 wt.%, (f) 10.0 wt.%.


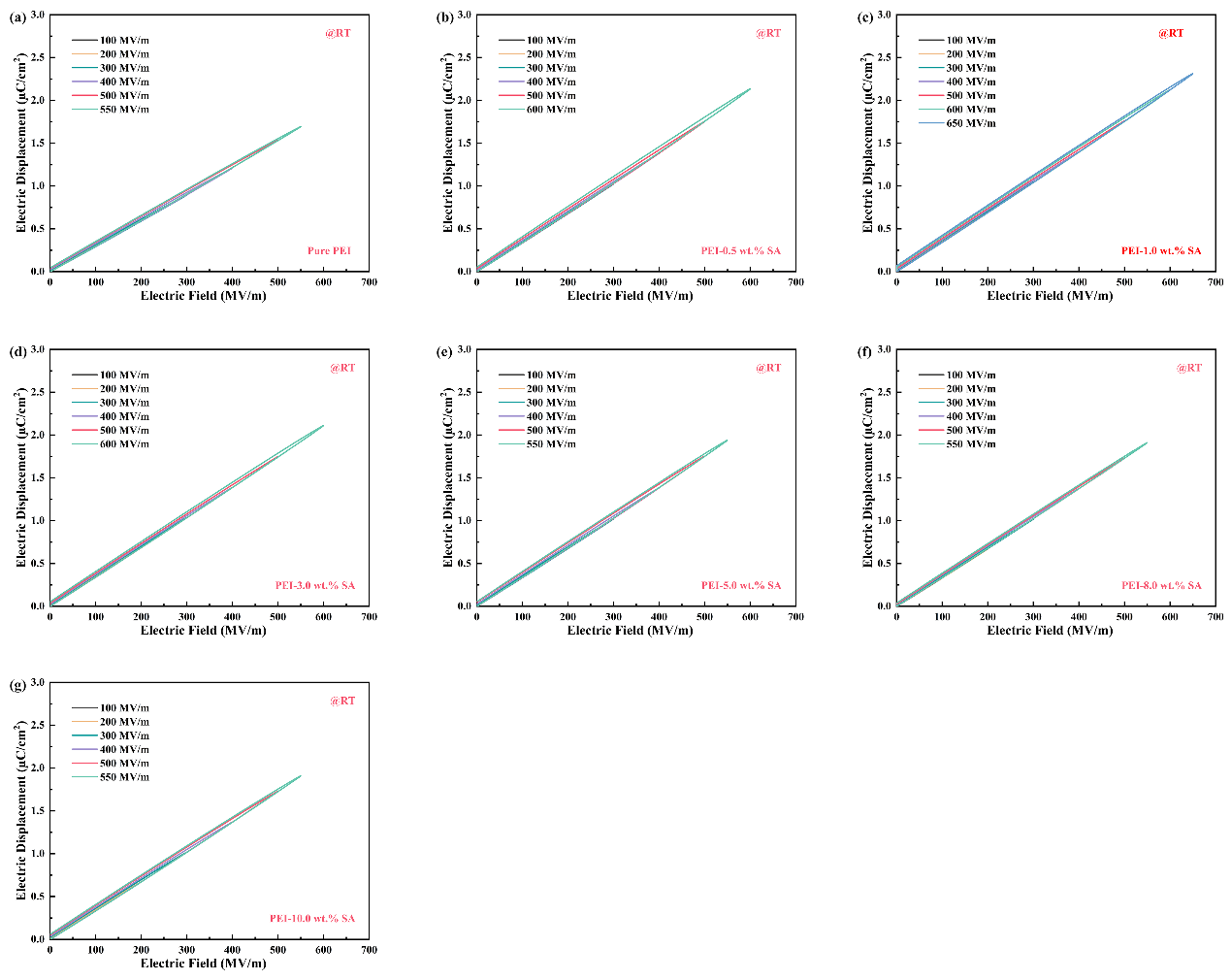


**Figure S31.** *D-E* loops of PEI-SA at room temperature: (a) Pure PEI, (b) 0.5 wt.%, (c) 1.0 wt.%, (d) 3.0 wt.%, (e) 5.0 wt.%, (f) 8.0 wt.%, (g) 10.0 wt.%.


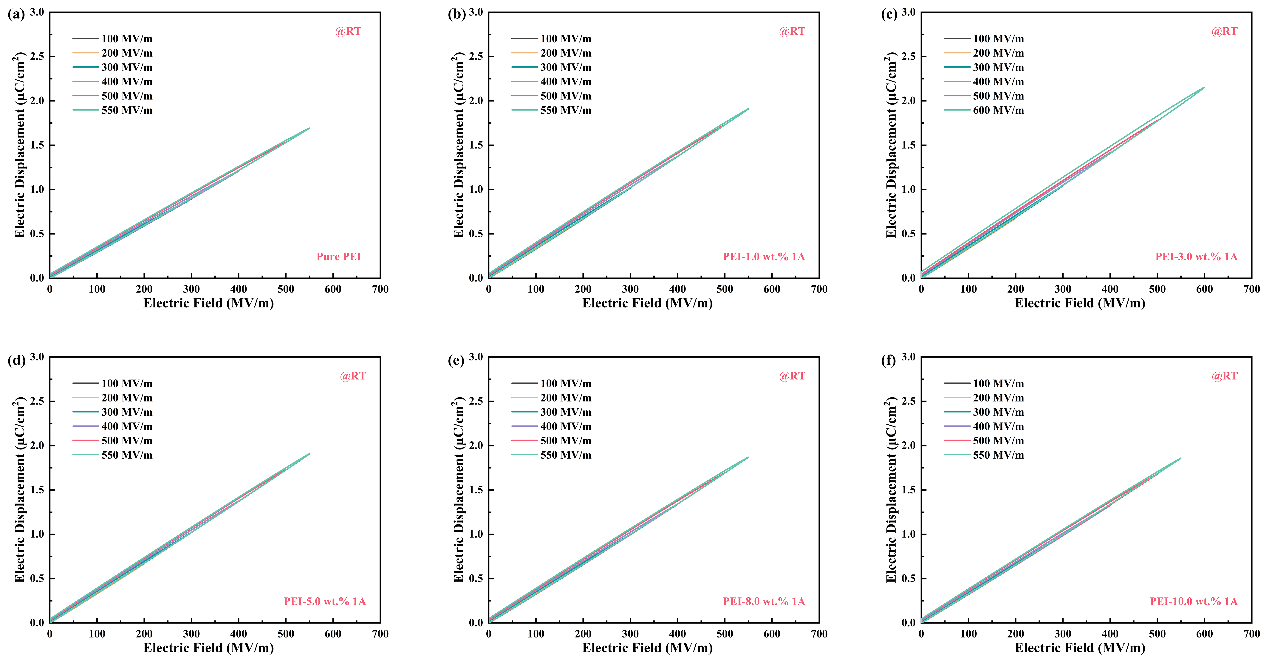


**Figure S32.** *D-E* loops of PEI-1A at room temperature: (a) Pure PEI, (b) 1.0 wt.%, (c) 3.0 wt.%, (d) 5.0 wt.%, (e) 8.0 wt.%, (f) 10.0 wt.%.


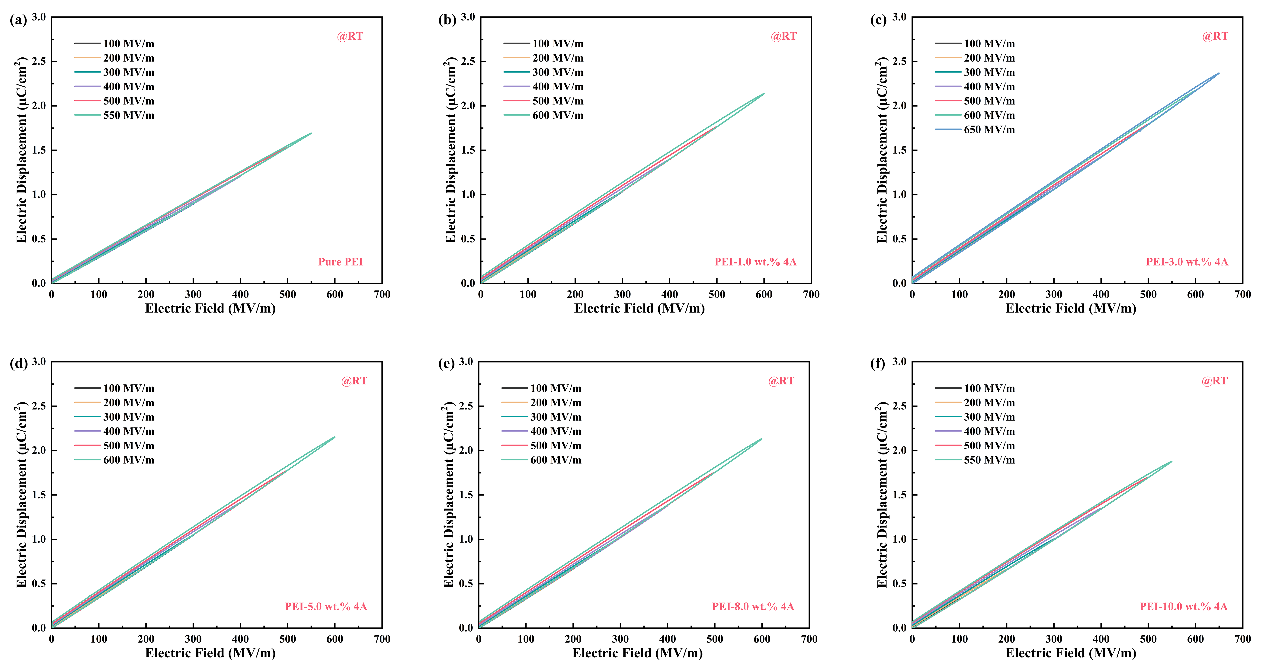


**Figure S33.** *D-E* loops of PEI-4A at room temperature: (a) Pure PEI, (b) 1.0 wt.%, (c) 3.0 wt.%, (d) 5.0 wt.%, (e) 8.0 wt.%, (f) 10.0 wt.%.


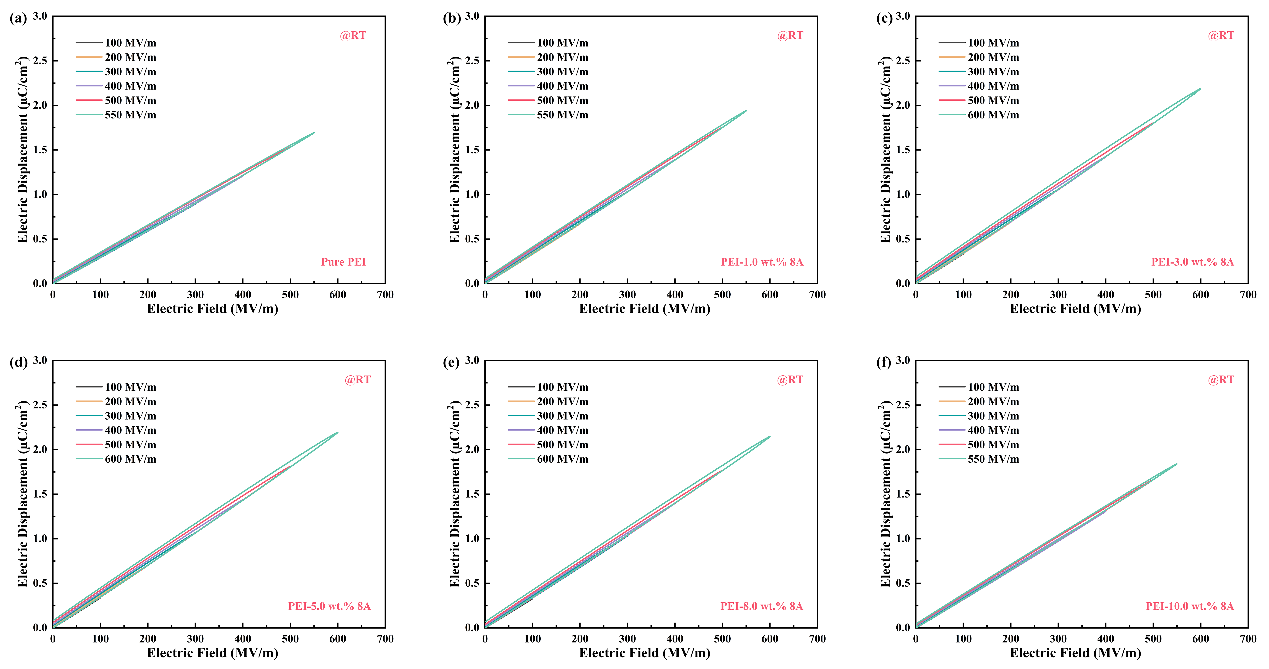


**Figure S34.** *D-E* loops of PEI-8A at room temperature: (a) Pure PEI, (b) 1.0 wt.%, (c) 3.0 wt.%, (d) 5.0 wt.%, (e) 8.0 wt.%, (f) 10.0 wt.%.


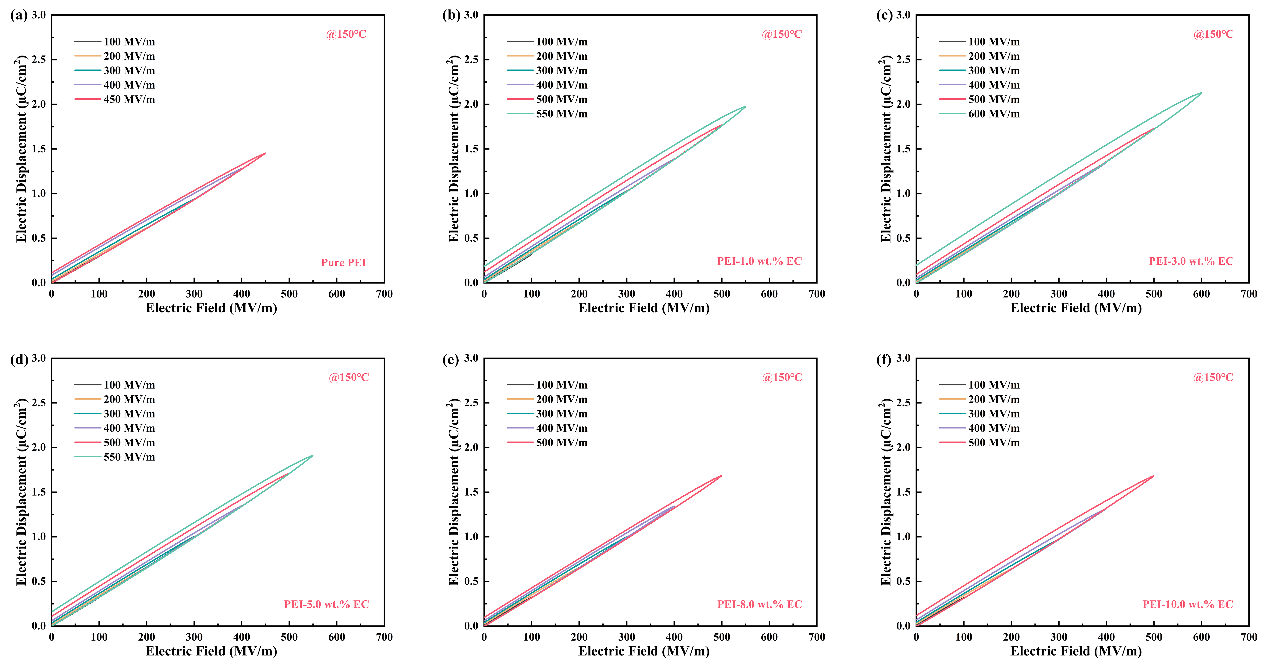


**Figure S35.** *D-E* loops of PEI-EC at 150 ℃: (a) Pure PEI, (b) 1.0 wt.%, (c) 3.0 wt.%, (d) 5.0 wt.%, (e) 8.0 wt.%, (f) 10.0 wt.%.


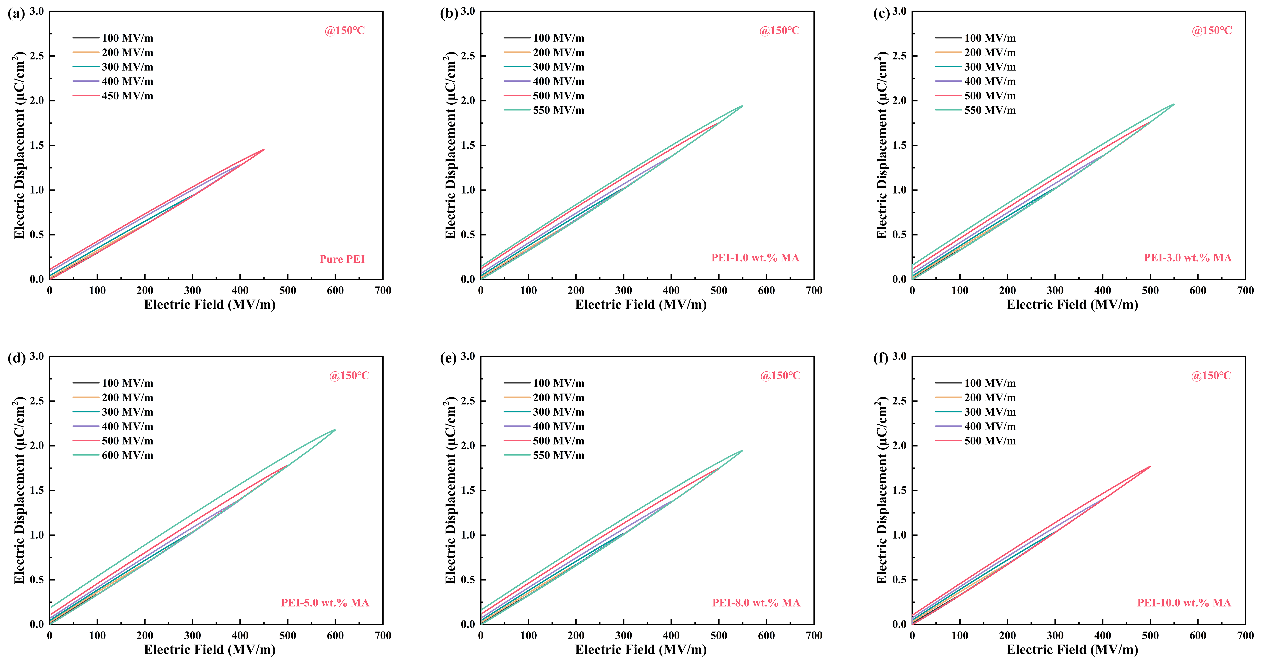


**Figure S36.** *D-E* loops of PEI-MA at 150 ℃: (a) Pure PEI, (b) 1.0 wt.%, (c) 3.0 wt.%, (d) 5.0 wt.%, (e) 8.0 wt.%, (f) 10.0 wt.%.

.


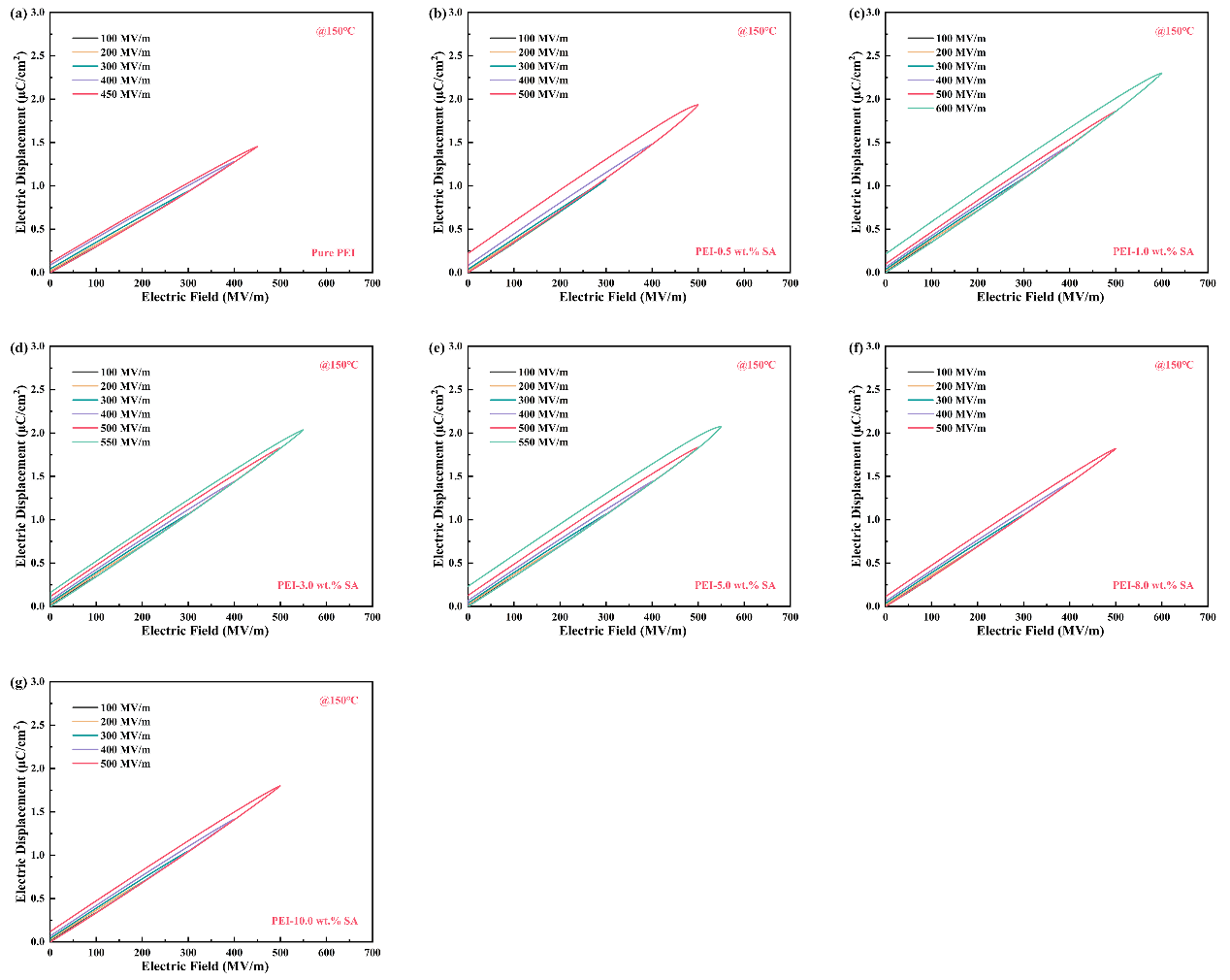


**Figure S37.** *D-E* loops of PEI-SA at 150 ℃: (a) Pure PEI, (b) 0.5 wt.%, (c) 1.0 wt.%, (d) 3.0 wt.%, (e) 5.0 wt.%, (f) 8.0 wt.%, (g) 10.0 wt.%.


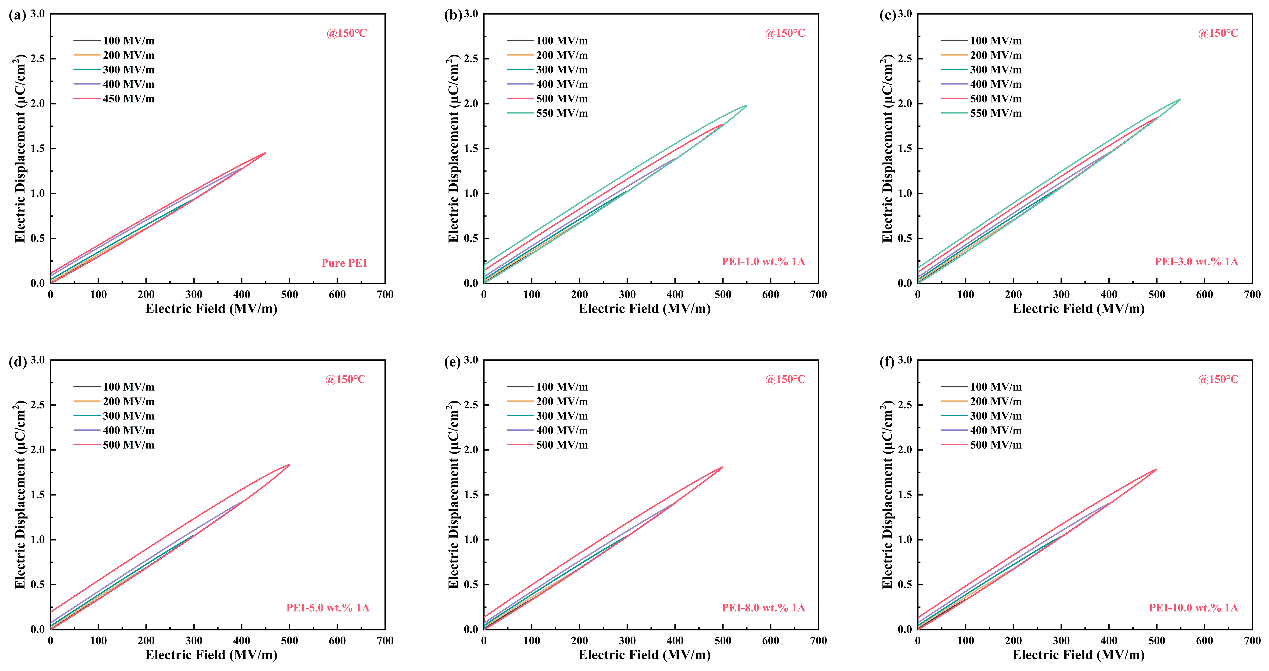


**Figure S38.** *D-E* loops of PEI-1A at 150 ℃: (a) Pure PEI, (b) 1.0 wt.%, (c) 3.0 wt.%, (d) 5.0 wt.%, (e) 8.0 wt.%, (f) 10.0 wt.%.


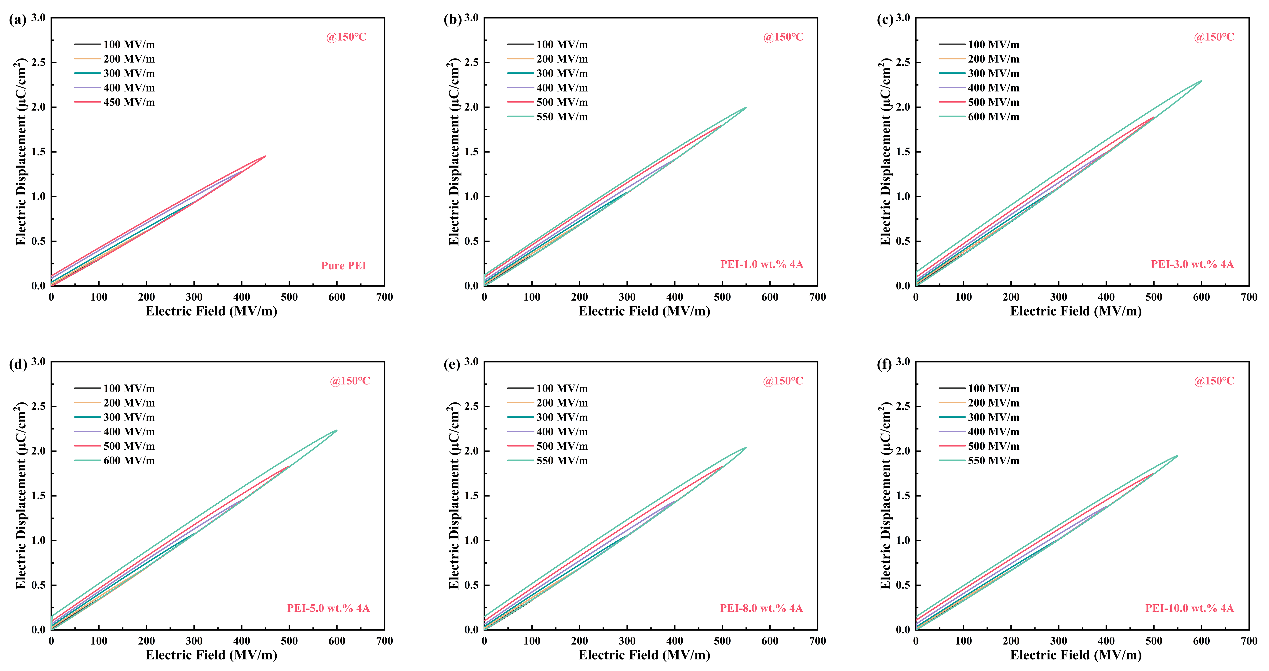


**Figure S39.** *D-E* loops of PEI-4A at 150 ℃: (a) Pure PEI, (b) 1.0 wt.%, (c) 3.0 wt.%, (d) 5.0 wt.%, (e) 8.0 wt.%, (f) 10.0 wt.%.


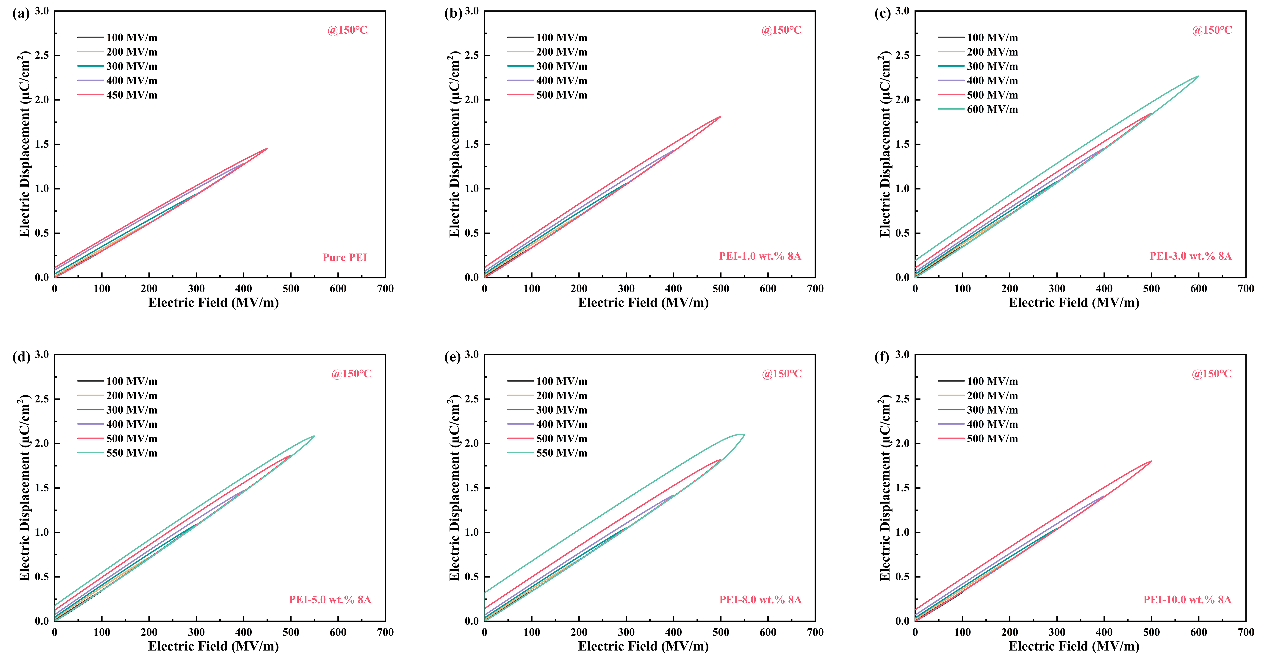


**Figure S40.** *D-E* loops of PEI-8A at 150 ℃: (a) Pure PEI, (b) 1.0 wt.%, (c) 3.0 wt.%, (d) 5.0 wt.%, (e) 8.0 wt.%, (f) 10.0 wt.%.


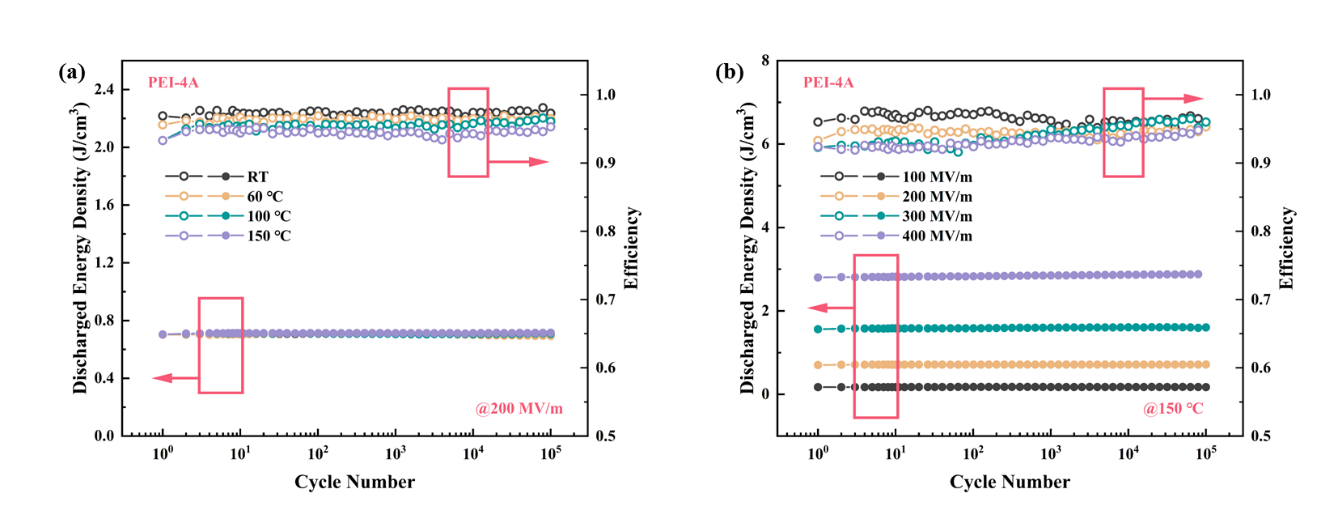


**Figure S41.** Discharged energy density and efficiency versus cycle number of PEI-3 wt.% 4A at (a) different temperatures and (b) different electric field.

**References**

[1] S. Li, D. Min, W. Wang, G. Chen, *ITDEI* **2016**, *23* (5), 2777, https://doi.org/10.1109/TDEI.2016.7736837.

[2] F. A. Sahki, A. Bouraiou, S. Taboukhat, L. Messaadia, S. Bouacida, V. Figa, K. Bouchouit, B. Sahraoui, *Optik* **2021**, *241*, 166949, https://doi.org/10.1016/j.ijleo.2021.166949.

[3] T. Lu, F. Chen, *J. Comput. Chem.* **2012**, *33* (5), 580, http://doi.org/10.1002/jcc.22885.

[4] J. Zhang, T. Lu, *PCCP* **2021**, *23* (36), 20323. http://doi.org/10.1039/D1CP02805G.

[5] M. Matsumoto, T. Nishimura, *TOMACS* **1998**, *8* (1), 3, https://doi.org/10.1145/272991.272995.

[6] N. Rakkapao, V. Vao-soongnern, *J. Polym. Res.* **2014**, *21*, 1, http://doi.org/10.1007/s10965-014-0606-1.

[7] Z-H. Shen, J.-J. Wang, Y. Lin, C.-W. Nan, L.-Q. Chen, Y. Shen, *Adv. Mater.* **2018**, *30* (2), 1704380, https://doi.org/https://doi.org/10.1002/adma.201704380.
